# Supplementary material for: Characterization of the literature informing health care of transgender and gender-diverse persons: A bibliometric analysis
Source: PLoS One. 2024 Oct 4;19(10):e0309169. doi: 10.1371/journal.pone.0309169 (PMC11452042; doi:10.1371/journal.pone.0309169)
Supplement: S1 File — (DOCX) [file pone.0309169.s001.docx]

**Table S1: Summary of study characteristics of studies across chapters 1-6.**

|  | Chapter 1  n = 1 | Chapter 2  n = 55 | Chapter 3  n = 22 | Chapter 4  n = 6 | Chapter 5  n = 63 | Chapter 6  n = 111 |
| --- | --- | --- | --- | --- | --- | --- |
| **Median Year**  **(Range)** | 2013  - | 2018  (2009 – 2022) | 2017  (2012 – 2021) | 2017  (2013 – 2021) | 2017  (2000 – 2021) | 2018  (1969 – 2022) |
| Study Design n (%)  Cross-sectional  Prospective Cohort Study  Retrospective Cohort Study  Qualitative  Mixed Methods  Randomized Controlled Trial  Case Control  Pre-post | 1 (100)  -  -  -  -  -  -  - | 28 (51)  5 (9)  6 (11)  7 (13)  7 (13)  -  1 (2)  1 (2) | 17 (77)  2 (14)  3 (9)  -  -  -  -  - | 3 (50)  1 (17)  -  1 (17)  -  1 (17)  -  - | 34 (54)  6 (10)  6 (10)  7 (11)  6 (10)  -  3 (5)  1 (2) | 49 (44)  28 (25)  9 (8)  11 (10)  6 (5)  3 (3)  1 (1)  4 (4) |
| **Gender Identities n (%)**  Cisgender  Eunuch  Intersex  LGBTQ  Not Reported  Questioning  TGD + Cisgender  TGD | -  -  -  -  -  -  -  1 (100) | -  -  -  1 (2)  1 (2)  1 (2)  4 (7)  48 (7) | -  -  -  -  3 (14)  5 (23)  6 (27)  8 (36) | 1 (17)  -  -  1 (17)  -  -  -  4 (66) | -  -  -  -  3 (4)  6 (10)  7 (11)  47 (75) | 1 (1)  -  -  6 (5)  14 (13)  1 (1)  13 (12)  51 (46) |
| **Race / Ethnicity**  **n (%) ***  Asian or Pacific Islander  Black or African Descent  Hispanic or Latinx  Indigenous, Aboriginal, or Native  Multiple Races/Ethnicities  Not Disclosed or Unknown  Not reported  Other  White | 1 (100)  -  1 (100)  -  -  -  -  -  1 (100) | 8 (15)  9 (16)  10 (18)  6 (11)  5 (9)  3 (5)  38 (69)  9 (25)  10 (18) | 5 (23)  9 (41)  8 (36)  2 (9)  1 (5)  7 (32)  12 (55)  7 (32)  9 (41) | 3 (50)  4 (67)  3 (50)  3 (50)  2 (33)  1 (17)  1 (17)  2 (33)  5 (83) | 18 (29)  18 (29)  14 (22)  7 (11)  11 (17)  9 (14)  35 (56)  6 (25)  25 (40) | 27 (24)  34 (31)  31 (28)  19 (17)  27 (24)  18 (16)  58 (52)  31 (28)  52 (47) |
| **Age Groups Represented**  **n (Years; %) ***  12 ≤ age  13 ≤ age ≤ 17  18 ≤ age ≤ 29  30 ≤ age ≤ 50  51 ≥ age | -  -  -  -  - | 2 (4)  14 (25)  40 (73)  35 (64)  23 (42) | 7 (32)  9 (41)  12 (55)  9 (41)  9 (41) | -  2 (33)  3 (50)  4 (67)  4 (67) | 4 (6)  17 (27)  37 (59)  33 (52)  24 (38) | 42 (38)  68 (61)  72 (65)  18 (16)  12 (11) |
| **Stratification by Gender n (%)**  Yes  No | -  1 (100) | 15 (27)  40 (73) | 10 (45)  12 (55) | 2 (33)  4 (67) | 25 (40)  38 (60) | 40 (36)  71 (64) |
| **Gender Included as a Covariate in Analysis n (%)**  Yes  No | -  1 (100) | 4 (7)  51 (93) | 1 (5)  21 (95) | -  6 (100) | 5 (8)  58 (92) | 11 (10)  100 (90) |
| **Stratification by Sex n (%)**  Yes  No | -  1 (100) | 15 (27)  40 (73) | 18 (82)  4 (18) | 2 (33)  4 (67) | 25 (40)  38 (60) | 49 (44)  62 (56) |
| **Sex Included as a Covariate in Analysis n (%)**  Yes  No | -  1 (100) | 2 (4)  53 (96) | 2 (9)  20 (91) | -  6 (100) | 1 (2)  62 (98) | 13 (12)  98 (88) |
| **Reported Disability and/or Chronic Illness**  Yes  No | 1 (100)  - | 20 (36)  35 (64) | 5 (23)  17 (77) | 3 (50)  3 (50) | 20 (32)  43 (68) | 21 (19)  90 (81) |
| **Reported Neurodiversity?**  Yes  No | -  1 (100) | 3 (5)  52 (95) | 1 (5)  21 (95) | -  6 (100) | 4 (6)  94 (59) | 14 (13)  97 (87) |

Notes. Chapter 1: Terminology. Chapter 2: Global Applicability. Chapter 3: Population Estimates. Chapter 4: Education. Chapter 5: Assessment of Adults. Chapter 6: Adolescents. LGBTQ: Lesbian, Queer, Bisexual, Transgender, and Queer. Questioning: Individuals who were questioning their gender identity. TGD: Transgender and Gender-Diverse. ***:** Percentages add up to more than 100% and n adds up to more than total number of studies due to multiple age groups and/or racial/ethnic groups being represented in some studies.

**Table S2. Summary of study characteristics of studies across chapters 7-13 (excluding chapter 12).**

|  | Chapter 7  n = 35 | Chapter 8  n = 45 | Chapter 9  n = 16 | Chapter 10  n = 34 | Chapter 11  n = 11 | Chapter 13  n = 80 |
| --- | --- | --- | --- | --- | --- | --- |
| **Median Year (Range)** | 2018  (1998 – 2022) | 2018  (1979 – 2022) | 2016  (2007 – 2021) | 2018  (2009 – 2023) | 2016  (2004 – 2020) | 2017  (1998 – 2022) |
| **Study Design n (%)**  Cross-sectional  Prospective Cohort Study  Retrospective Cohort Study  Qualitative  Mixed Methods  Randomized Controlled Trial  Case Control  Pre-post | 13 (37)  10 (29)  -  7 (20)  3 (9)  -  1 (3)  1 (3) | 25 (56)  4 (9)  1 (2)  12 (27)  1 (2)  -  1 (2)  1 (2) | 8 (50)  -  -  2 (13)  1 (6)  4 (25)  -  1 (6) | 15 (44)  1 (3)  3 (9)  9 (26)  6 (18)  -  -  - | 4 (36)  -  -  5 (45)  1 (9)  -  1 (9)  - | 29 (36)  22 (28)  23 (29)  -  4 (5)  -  1 (1)  1 (1) |
| **Gender Identities n (%)**  Cisgender  Eunuch  Intersex  LGBTQ  Not Reported  Questioning  TGD + Cisgender  TGD | -  -  -  2 (6)  4 (11)  6 (17)  7 (20)  16 (46) | -  3 (7)  -  5 (11)  1 (2)  1 (2)  2 (4)  33 (73) | -  6 (38)  -  -  7 (44)  -  -  3 (19) | -  -  7 (21)  1 (3)  18 (53)  3 (9)  1 (3)  4 (12) | -  -  -  1 (9)  2 (18)  1 (9)  -  7 (64) | -  -  -  -  1 (1)  4 (5)  2 (3)  73 (91) |
| **Race / Ethnicity**  **n (%) ***  Asian or Pacific Islander  Black or African Descent  Hispanic or Latinx  Indigenous, Aboriginal, or Native  Multiple Races/Ethnicities  Not Disclosed or Unknown  Not reported  Other  White | 13 (37)  12 (34)  14 (40)  6 (17)  14 (40)  6 (17)  14 (40)  10 (29)  18 (51) | 15 (33)  21 (47)  16 (36)  6 (13)  16 (36)  8 (18)  20 (44)  8 (18)  23 (51) | -  -  -  -  -  1 (6)  14 (88)  1 (6)  1 (6) | 4 (13)  8 (24)  6 (18)  2 (6)  3 (9)  1 (3)  23 (65)  3 (9)  11 (32) | 4 (36)  5 (45)  3 (27)  -  3 (27)  5 (45)  5 (45)  3 (27)  6 (55) | 4 (5)  4 (5)  3 (4)  1 (1)  3 (4)  4 (5)  74 (93)  4 (5)  6 (8) |
| **Age Groups Represented**  **n (Years; %) ***  12 ≤ age  13 ≤ age ≤ 17  18 ≤ age ≤ 29  30 ≤ age ≤ 50  51 ≥ age | 19 (54)  22 (63)  17 (49)  4 (11)  2 (6) | 4 (9)  16 (36)  27 (60)  18 (40)  11 (24) | -  1 (6)  4 (25)  5 (31)  5 (31) | 6 (18)  10 (29)  20 (59)  16 (47)  13 (38) | 4 (36)  1 (9)  4 (36)  3 (27)  3 (27) | 1 (1)  13 (16)  57 (71)  59 (74)  37 (46) |
| **Stratification by Gender n (%)**  Yes  No | 10 (29)  25 (71) | 16 (36)  29 (64) | 2 (12)  14 (88) | 2 (6)  32 (94) | 1 (9)  10 (91) | 10 (12)  70 (88) |
| **Gender Included as a Covariate in Analysis n (%)**  Yes  No | 3 (9)  32 (91) | 1 (2)  44 (98) | -  16 (100) | -  34 (100) | -  11 (100) | -  80 (100) |
| **Stratification by Sex n (%)**  Yes  No | 15 (43)  20 (57) | 17 (38)  28 (62) | 5 (31)  11 (69) | 8 (24)  26 (76) | 1 (9)  10 (91) | 12 (15)  68 (85) |
| **Sex Included as a Covariate in Analysis n (%)**  Yes  No | 6 (17)  29 (83) | 1 (2)  44 (98) | -  16 (100) | -  34 (100) | -  11 (100) | -  80 (100) |
| **Reported Disability and/or Chronic Illness**  Yes  No | 4 (11)  31 (89) | 8 (18)  37 (82) | 3 (19)  13 (81) | 6 (18)  28 (82) | 4 (36)  7 (64) | 15 (19)  65 (81) |
| **Reported Neurodiversity?**  Yes  No | 5 (14)  30 (86) | 1 (2)  44 (98) | -  16 (100) | 2 (6)  32 (94) | -  11 (100) | 1 (1)  79 (99) |

Notes. Chapter 7: Children. Chapter 8: Nonbinary. Chapter 9: Eunuchs. Chapter 10: Intersex. Chapter 11: Institutional Environments. Chapter 13: Surgery and Postoperative Care. LGBTQ: Lesbian, Queer, Bisexual, Transgender, and Queer. Questioning: Individuals who were questioning their gender identity. TGD: Transgender and Gender-Diverse. ***:** Percentages add up to more than 100% and n adds up to more than total number of studies due to multiple age groups and/or racial/ethnic groups being represented in some studies.

**Table S3: Summary of study characteristics of studies across chapters 14-18.**

|  | Chapter 14  n = 55 | Chapter 15  n = 55 | Chapter 16  n = 56 | Chapter 17  n = 48 | Chapter 18  n = 54 |
| --- | --- | --- | --- | --- | --- |
| **Median Year (Range)** | 2014  (1999 – 2022) | 2018  (2004 – 2022) | 2018  (1985 – 2021) | 2019  (2008 – 2023) | 2017  (2000 – 2021) |
| Study Design n (%)  Cross-sectional  Prospective Cohort Study  Retrospective Cohort Study  Qualitative  Mixed Methods  Randomized Controlled Trial  Case Control  Pre-post | 13 (24)  15 (27)  18 (33)  3 (5)  3 (5)  2 (4)  1 (2)  - | 36 (65)  6 (11)  8 (15)  4 (7)  -  -  1 (2)  - | 26 (46)  7 (13)  8 (14)  9 (16)  5 (9)  -  -  1 (2) | 25 (52)  6 (13)  -  12 (25)  4 (8)  -  -  1 (2) | 36 (67)  4 (7)  4 (7)  4 (7)  2 (4)  1 (2)  5 (9)  1 (2) |
| **Gender Identities n (%)**  Cisgender  Eunuch  Intersex  LGBTQ  Not Reported  Questioning  TGD + Cisgender  TGD | -  -  -  -  -  -  6 (11)  49 (89) | -  -  -  3 (5)  3 (5)  -  15 (27)  34 (62) | -  -  -  3 (5)  13 (23)  -  13 (23)  36 (64) | -  -  -  2 (4)  3 (6)  -  6 (13)  37 (77) | -  -  -  4 (7)  6 (11)  1 (2)  6 (11)  37 (69) |
| **Race / Ethnicity**  **n (%) ***  Asian or Pacific Islander  Black or African Descent  Hispanic or Latinx  Indigenous, Aboriginal, or Native  Multiple Races/Ethnicities  Not Disclosed or Unknown  Not reported  Other  White | 4 (7)  6 (11)  5 (9)  1 (2)  4 (7)  1 (2)  49 (89)  2 (4)  5 (9) | 15 (27)  34 (62)  26 (47)  10 (18)  15 (27)  13 (24)  18 (32)  22 (40)  36 (65) | 13 (23)  14 (25)  12 (21)  5 (9)  7 (13)  10 (18)  32 (57)  12 (21)  22 (39) | 11 (23)  14 (29)  15 (31)  7 (15)  8 (17)  7 (15)  28 (58)  14 (29)  18 (38) | 17 (31)  21 (39)  18 (33)  9 (17)  10 (19)  9 (17)  20 (37)  21 (39)  31 (57) |
| **Age Groups Represented**  **n (Years; %) ***  12 ≤ age  13 ≤ age ≤ 17  18 ≤ age ≤ 29  30 ≤ age ≤ 50  51 ≥ age | -  4 (7)  40 (73)  47 (85)  33 (60) | 1 (2)  2 (4)  36 (65)  40 (73)  37 (67) | 8 (14)  21 (38)  41 (73)  31 (55)  14 (25) | 3 (6)  9 (19)  31 (65)  24 (50)  19 (40) | 2 (4)  13 (24)  38 (70)  36 (67)  29 (54) |
| **Stratification by Gender n (%)**  Yes  No | 7 (13)  48 (87) | 30 (55)  25 (45) | 10 (18)  46 (82) | 18 (37)  30 (63) | 21 (39)  33 (61) |
| **Gender Included as a Covariate in Analysis n (%)**  Yes  No | -  55 (100) | 1 (2)  54 (98) | -  56 (100) | -  48 (100) | 5 (9)  49 (91) |
| **Stratification by Sex n (%)**  Yes  No | 7 (13)  48 (87) | 26 (47)  29 (53) | 10 (18)  46 (82) | 18 (37)  30 (63) | 19 (35)  35 (65) |
| **Sex Included as a Covariate in Analysis n (%)**  Yes  No | -  55 (100) | 4 (7)  51 (93) | -  56 (100) | -  48 (100) | 2 (4)  52 (96) |
| **Reported Disability and/or Chronic Illness**  Yes  No | -  55 (100) | 20 (36)  35 (64) | 8 (14)  48 (86) | 14 (29)  34 (71) | 24 (44)  30 (56) |
| **Reported Neurodiversity?**  Yes  No | -  55 (100) | 1 (2)  54 (98) | 1 (2)  55 (98) | -  48 (100) | 4 (7)  50 (93) |

Notes. Chapter 15: Primary Care. Chapter 16: Reproductive Health. Chapter 17: Sexual Health. Chapter 18: Mental Health. TGD: Transgender and Gender-Diverse. LGBTQ: Lesbian, Queer, Bisexual, Transgender, and Queer. Questioning: Individuals who were questioning their gender identity. TGD: Transgender and Gender-Diverse. *: Percentages add up to more than 100% and n adds up to more than total number of studies due to multiple age groups and/or racial/ethnic groups being represented in some studies.
