# Supplementary material for: Characterization of the literature informing health care of transgender and gender-diverse persons: A bibliometric analysis
Source: PLoS One. 2024 Oct 4;19(10):e0309169. doi: 10.1371/journal.pone.0309169 (PMC11452042; doi:10.1371/journal.pone.0309169)
Supplement: Attachment — Submitted filename: PONE-D-24-13832_reviewer.pdf [file pone.0309169.s003.pdf]

PLOS ONE

Characterization of the literature informing health Care of transgender and gender-diverse persons: A bibliometric analysis

--Manuscript Draft--

**Manuscript Number:**	PONE-D-24-13832
**Article Type:**	Research Article
**Full Title:**	Characterization of the literature informing health Care of transgender and gender-diverse persons: A bibliometric analysis
**Short Title:**	The state of literature informing TGD care
**Corresponding Author:**	Sofia Ahmed, MD, MMSc<br>University of Alberta Faculty of Medicine & Dentistry<br>Edmonton, Alberta CANADA
**Keywords:**	Transgender<br>Gender-Diverse<br>Cisgender<br>Representation<br>Gender-Affirming Hormone Therapy
**Abstract:**	Background and objective<br>Transgender and gender-diverse (TGD) persons experience health inequities compared to their cisgender peers, which is in part related to limited evidence informing their care. Thus, we aimed to characterize the literature informing care provision of TGD individuals.<br>Data source, eligibility criteria, and synthesis methods<br>Literature cited by the World Professional Association of Transgender Health Standards of Care Version 8 was reviewed. Original research articles, excluding systematic reviews (n=74), were assessed (n=1809). Studies where the population of interest were only caregivers, providers, siblings, partners, or children of TGD individuals were excluded (n=7). Results were synthesized in a descriptive manner.<br>Results<br>Of 1809 citations, 696 studies met the inclusion criteria. TGD-only populations were represented in 65% of studies. White (38%) participants and young adults (18 to 29 years old, 64%) were the most well-represented study populations. Almost half of studies (45%) were cross-sectional in nature. Overall, the median number of TGD participants (median [IQR]: 104 [32, 356]) included in each study was approximately one third of included cisgender participants (271 [47, 15405]). In studies where both TGD and cisgender individuals were included (n=74), the proportion of TGD to cisgender participants was 1:2 [1:20, 1:1]. Less than a third of studies stratified results by sex (32%) or gender (28%), and even fewer included sex (4%) or gender (3%) as a covariate in the analysis. The proportion of studies with populations including both TGD and cisgender participants increased between 1969 and 2023, while the proportion of studies with study populations of unspecified gender identity decreased over the same time period.<br>Conclusions<br>While TGD participant-only studies make up most of the literature informing care of this population, longitudinal studies including a diversity of TGD individuals across life stages are required to improve the quality of evidence.
**Order of Authors:**	Badal S.B. Pattar, BSc
	Nabilah Gulamhusein, BSc
	Chantal L. Rytz, MSc
	Keila Turino Miranda, MSc
	Lauren B. Beach, JD, PhD
	Zack Marshall, PhD
	David Collister, MD, PhD

Powered by Editorial Manager® and ProduXion Manager® from Aries Systems Corporation

	Dina N. Greene, PhD
	Cameron T. Whitley, PhD
	Nathalie Saad, MD, MSc
	Sandra M. Dumanski, MD, MSc
	Tyrone G. Harrison, MD, PhD
	Lindsay Peace, BA
	Ameila M. Newbert
	Sofia B. Ahmed, MD, MMSc
**Additional Information:**	
**Question**	**Response**
**Financial Disclosure**<br><br>Enter a financial disclosure statement that describes the sources of funding for the work included in this submission. Review the submission guidelines for detailed requirements. View published research articles from *PLOS ONE* for specific examples.<br><br>This statement is required for submission and **will appear in the published article** if the submission is accepted. Please make sure it is accurate.<br><br>**Funded studies**<br>Enter a statement with the following details:<br>• Initials of the authors who received each award<br>• Grant numbers awarded to each author<br>• The full name of each funder<br>• URL of each funder website<br>• Did the sponsors or funders play any role in the study design, data collection and analysis, decision to publish, or preparation of the manuscript?<br><br>Did you receive funding for this work?	The author(s) received no specific funding for this work.
**Competing Interests**<br><br>Use the instructions below to enter a competing interest statement for this submission. On behalf of all authors, disclose any competing interests that could be perceived to bias this	The authors have declared that no competing interests exist.

work—acknowledging all financial support and any other relevant financial or non-financial competing interests.<br><br>This statement is required for submission and **will appear in the published article** if the submission is accepted. Please make sure it is accurate and that any funding sources listed in your Funding Information later in the submission form are also declared in your Financial Disclosure statement.<br><br>View published research articles from *PLOS ONE* for specific examples.<br><br>**NO authors have competing interests**<br><br>Enter: *The authors have declared that no competing interests exist.*<br><br>**Authors with competing interests**<br><br>Enter competing interest details beginning with this statement:<br><br>*I have read the journal's policy and the authors of this manuscript have the following competing interests: [insert competing interests here]*<br><br>* typeset	
**Ethics Statement**<br><br>Enter an ethics statement for this submission. This statement is required if the study involved:<br><br>• Human participants<br>• Human specimens or tissue<br>• Vertebrate animals or cephalopods<br>• Vertebrate embryos or tissues<br>• Field research<br><br>Write "N/A" if the submission does not require an ethics statement.	Institutional review board approval was not required as this study used only secondary, published data.

Powered by Editorial Manager® and ProduXion Manager® from Aries Systems Corporation

General guidance is provided below. Consult the submission guidelines for detailed instructions. **Make sure that all information entered here is included in the Methods section of the manuscript.**<br><br>**Format for specific study types**<br><br>**Human Subject Research (involving human participants and/or tissue)**<br>• Give the name of the institutional review board or ethics committee that approved the study<br>• Include the approval number and/or a statement indicating approval of this research<br>• Indicate the form of consent obtained (written/oral) or the reason that consent was not obtained (e.g. the data were analyzed anonymously)<br><br>**Animal Research (involving vertebrate animals, embryos or tissues)**<br>• Provide the name of the Institutional Animal Care and Use Committee (IACUC) or other relevant ethics board that reviewed the study protocol, and indicate whether they approved this research or granted a formal waiver of ethical approval<br>• Include an approval number if one was obtained<br>• If the study involved *non-human primates*, add *additional details* about animal welfare and steps taken to ameliorate suffering<br>• If anesthesia, euthanasia, or any kind of animal sacrifice is part of the study, include briefly which substances and/or methods were applied<br><br>**Field Research**<br><br>Include the following details if this study involves the collection of plant, animal, or other materials from a natural setting:<br>• Field permit number<br>• Name of the institution or relevant body that granted permission	
**Data Availability**<br><br>Authors are required to make all data underlying the findings described fully	No - some restrictions will apply

available, without restriction, and from the time of publication. PLOS allows rare exceptions to address legal and ethical concerns. See the PLOS Data Policy and FAQ for detailed information.<br><br>A Data Availability Statement describing where the data can be found is required at submission. Your answers to this question constitute the Data Availability Statement and **will be published in the article**, if accepted.<br><br>**Important:** Stating 'data available on request from the author' is not sufficient. If your data are only available upon request, select 'No' for the first question and explain your exceptional situation in the text box.<br><br>Do the authors confirm that all data underlying the findings described in their manuscript are fully available without restriction?	
**Describe where the data may be found in full sentences. If you are copying our sample text, replace any instances of XXX with the appropriate details.**<br><br>• If the data are **held or will be held in a public repository**, include URLs, accession numbers or DOIs. If this information will only be available after acceptance, indicate this by ticking the box below. For example: *All XXX files are available from the XXX database (accession number(s) XXX, XXX.).*<br>• If the data are all contained **within the manuscript and/or Supporting Information files**, enter the following: *All relevant data are within the manuscript and its Supporting Information files.*<br>• If neither of these applies but you are able to provide **details of access elsewhere**, with or without limitations, please do so. For example:<br><br>*Data cannot be shared publicly because of [XXX]. Data are available from the XXX Institutional Data Access / Ethics*	The data underlying the results presented in the study are available from the World Professional Association of Transgender Health (URL: https://www.wpath.org/soc8/chapters).

Powered by Editorial Manager® and ProduXion Manager® from Aries Systems Corporation

Committee (contact via XXX) for researchers who meet the criteria for access to confidential data.<br><br>*The data underlying the results presented in the study are available from (include the name of the third party and contact information or URL).*<br>• This text is appropriate if the data are owned by a third party and authors do not have permission to share the data.<br><br>* typeset	
Additional data availability information:	Tick here if the URLs/accession numbers/DOIs will be available only after acceptance of the manuscript for publication so that we can ensure their inclusion before publication.

Powered by Editorial Manager® and ProduXion Manager® from Aries Systems Corporation

Manuscript

Click here to access/download;Manuscript;Manuscript Pattar et al.docx

Full Title: Characterization of the literature informing health Care of transgender and gender-diverse persons: A bibliometric analysis

Authors: Badal S.B. Pattar[1-3], Nabilah Gulamhusein[1-3], Chantal L. Rytz[1-3], Keila Turino Miranda[4], Lauren B. Beach[5], Zack Marshall[1,3], David Collister[6], Dina N. Greene[7], Cameron T. Whitley[8], Nathalie Saad[1,9], Sandra M. Dumanski[1-3,9], Tyrone G. Harrison[1-3,9], Lindsay Peace[10], Amelia M. Newbert[10], Sofia B. Ahmed[2,6]*

Affiliations: [1]Cumming School of Medicine, University of Calgary, Calgary, Alberta, Canada, [2]Libin Cardiovascular Institute, University of Calgary, Calgary, Alberta, Canada, [3]O'Brien Institute for Public Health, University of Calgary, Calgary, Alberta, Canada, [4]McGill University, Montreal, Quebec, Canada, [5]Department of Medical Social Sciences, Northwestern Feinberg School of Medicine, Chicago, Illinois, Unites States of America, [6]Department of Medicine, University of Alberta, Edmonton, Alberta, Canada, [7]Department of Laboratory Medicine, University of Washington, Seattle, Washington, Unites States of America, [8]Department of Sociology, Western Washington University, Bellingham, Washington, United States of America, [9]Department of Medicine, University of Calgary, Calgary, Alberta, Canada, [10]Skipping Stone Foundation, Calgary, Alberta, Canada

Correspondence: Sofia B. Ahmed, MD, MMSc, FRCPC
11-135 Clinical Sciences Building, 11220 83 Ave NW
Edmonton, Alberta, Canada, T6G 2B7
Email: Sofia.Ahmed@albertahealthservices.ca

Abstract

Background and objective

Transgender and gender-diverse (TGD) persons experience health inequities compared to their cisgender peers, which is in part related to limited evidence informing their care. Thus, we aimed to characterize the literature informing care provision of TGD individuals.

Data source, eligibility criteria, and synthesis methods

Literature cited by the World Professional Association of Transgender Health Standards of Care Version 8 was reviewed. Original research articles, excluding systematic reviews (n=74), were assessed (n=1809). Studies where the population of interest were only caregivers, providers, siblings, partners, or children of TGD individuals were excluded (n=7). Results were synthesized in a descriptive manner.

Results

Of 1809 citations, 696 studies met the inclusion criteria. TGD-only populations were represented in 65% of studies. White (38%) participants and young adults (18 to 29 years old, 64%) were the most well-represented study populations. Almost half of studies (45%) were cross-sectional in nature. Overall, the median number of TGD participants (median [IQR]: 104 [32, 356]) included in each study was approximately one third of included cisgender participants (271 [47, 15405]). In studies where both TGD and cisgender individuals were included (n=74), the proportion of TGD to cisgender participants was 1:2 [1:20, 1:1]. Less than a third of studies stratified results by sex (32%) or gender (28%), and even fewer included sex (4%) or gender (3%) as a covariate in the analysis. The

proportion of studies with populations including both TGD and cisgender participants increased between 1969 and 2023, while the proportion of studies with study populations of unspecified gender identity decreased over the same time period.

Conclusions

While TGD participant-only studies make up most of the literature informing care of this population, longitudinal studies including a diversity of TGD individuals across life stages are required to improve the quality of evidence.

Introduction

The United States Preventive Services Task Force (USPSTF) has recently recognized the limited evidence informing the preventive care of populations based on gender identity; a catalog of current USPSTF suggestions revealed insufficient evidence to make any specific recommendations for transgender and gender diverse (TGD) populations (1). Underscoring the critical nature of literature informing the optimal care of TGD persons, a 2023 national cohort study found that TGD individuals in a universal health care system have a risk of all-cause mortality that is between 1.34 and 1.75 times greater than that of cisgender people (2).

The vital importance of diverse representation in study participants to optimize health outcomes has been previously highlighted (3), with calls for greater inclusion of participants from gender minorities in clinical research (4, 5). A recent report from the National Academies of Sciences, Engineering, and Medicine (6) underscores the impact of inequitable representation, leading to compromised generalizability of clinical research findings, increased health system costs, and lack of access to effective medical interventions; all of these complications compound intersectional health disparities in the populations currently underrepresented or excluded in clinical trials and research. Historically, there has been insufficient representation of women and minoritized populations in research, but representation has steadily increased over the last several decades at least in part due to changes in policies (7, 8) and law (9).

In 2017, ClinicalTrials.gov introduced an optional gender eligibility description data field (10). While only 25% of entries contained gender identity terms with a range of frequencies (11), this represents a promising step towards increasing TGD representation

in research. Overall, there has been increasing recognition of sex and gender as key variables in research, and the importance of representation of diverse populations in health research. Thus, we conducted a bibliometric analysis of the World Professional Association of Transgender Health Standards of Care Version 8 (WPATH SOC 8) (12), a comprehensive guideline based on the best available science and expert professional consensus for the health of TGD individuals. Our primary objective was to characterize the demographics and study methods of influential literature informing the care of TGD persons, with a specific secondary objective to explore gender-affirming hormone therapy guidelines.

Material and methods

Data source and searches

The reference list from WPATH SOC 8 was used as a proxy for influential literature related to care of TGD persons, which consists of 18 chapters that each focus on a specific aspect of TGD health care. All citations from WPATH SOC 8 were manually exported from each chapter and organized into 18 groups in EndNote (Version 9.3.3, Clarivate, London, United Kingdom).

Study selection

All published primary research articles were included. Systematic reviews (n = 74) were excluded to avoid "double-counting participants", as were narrative reviews (n = 232) commentaries (n = 103), editorials (n = 30). Studies where the population of interest were only caregivers, providers, siblings, and partners (n = 6), or children of TGD individuals (n = 1) were also excluded. Articles in languages other than English were translated to

English using Google Translate (13). Two reviewers (BSBP, NG) independently extracted data using a standardized data abstraction form in the Covidence platform (Cochrane Technology, Melbourne, Victoria, Australia). Extracted study characteristics included the publication year, the chapter(s) in which the reference was cited, study design, gender (e.g., TGD, cisgender, TGD and cisgender, not reported, etc.), race/ethnicity, and age composition of study population, sample size, proportion of TGD to cisgender individuals if these populations were included in the study, whether the final results were stratified by sex and/or gender, and whether sex and/or gender were included as a covariate in the study analysis. A quality assurance exercise was employed following extraction, where a random sample of 10% of all included articles were assessed in duplicate for consistency. Agreement between the two reviewers was 70% and discrepancies were resolved by a third reviewer (SBA).

Statistical analysis

Results were synthesized in a descriptive manner. Data were presented as medians and range for the entirety of the WPATH SOC 8, as well as by chapter, including the literature informing Chapter 12 “Hormone Therapy”. The linear-by-linear trend test (14) was used to determine temporal patterns in the gender identity of included participants, where a significant change in trend was indicated by $p<0.05$. All analyses were performed using Stata (Version 18, StataCorp, College, Texas, United States of America).

Results

Of 1809 cited articles, 696 studies met the inclusion criteria (**Fig 1**). The median year of publication was 2018 (range: 1969 to 2023) (**Table 1**). Although WPATH SOC 8 was published in 2022, 3 included articles were listed as "in press" and later published in 2023. TGD-only populations were represented in 65% of studies (**Fig 2)**, with young adults (18 to 29 years old, 64%) being the most well-represented group. Race/ethnicity was not reported in 59% of studies. Of the studies reporting race/ethnicity, white participants were the most represented (38%) (median per study [interquartile range (IQR)]) (130 [38, 640]), followed by Black or African descent (29%, 13 [3, 167]), Hispanic/Latinx (25%, 43 [6, 246]), and Asian/Pacific Islander (22%, 9 [3, 77]) participants. The most common study design was cross-sectional (45%), followed by prospective cohort (17%), retrospective cohort (14%), and qualitative (13%). Overall, the number of TGD participants per study (median [interquartile range (IQR)]) (104 [32, 356]) was approximately one third of cisgender participants (271 [47, 15405]). Less than one third of studies stratified results by sex (32%) or gender (28%) and even fewer included sex (4%) or gender (3%) as a covariate in analyses (data not shown). In studies where both TGD and cisgender individuals were included (n=74), the median proportion of TGD to cisgender participants was 1:2 [1:20, 1:1]. Data for individual chapters, excluding Chapter 12, are listed in **Supplementary File 1.**

Fig 1. Preferred Reporting Items for Systematic Reviews and Meta-Analyses (PRISMA) 2020 flow of studies. TGD: Transgender and Gender-Diverse. WPATH SOC 8: World Professional Association of Transgender Health Standards of Care Version 8.

Table 1. Summary of study characteristics of studies across all 18 chapters.

	All<br>n = 696 (100%)	Cross Sectional<br>n = 312 (45%)	Prospective Cohort<br>n = 120 (17%)	Retrospective Cohort<br>n = 92 (14%)	Qualitative<br>n = 91 (13%)	Mixed-Methods<br>n = 39 (5%)	Randomized Controlled Trial<br>n = 19 (3%)	Case Control<br>n = 13 (2%)	Pre-Post<br>n = 10 (1%)
**Median Year (Range)**	2018 (1969 –2023)	2018 (1999 – 2023)	2017 (1969 – 2022)	2018 (1997 – 2022)	2018 (2008 – 2023)	2016 (2000 – 2022)	2019 (2003 – 2021)	2018 (2006 – 2021)	2016 (1997 – 2020)
**Gender Identities n (%)**									
Cisgender	9 (1)	1 (<1)	2 (2)	1 (1)	-	-	2 (11)	3 (23)	-
Eunuch	6 (1)	4 (1)	-	-	1 (1)	1 (3)	-	-	-
Intersex	7 (1)	2 (<1)	1 (<1)	-	2 (2)	2 (5)	-	-	-
LGBTQ	27 (4)	12 (4)	-	1 (1)	9 (10)	4 (10)	-	-	1 (10)
Not Reported	80 (11)	32 (10)	22 (18)	7 (8)	10 (11)	2 (5)	5 (26)	-	2 (20)
Questioning	43 (6)	16 (5)	18 (15)	2 (2)	3 (3)	2 (5)	1 (5)	1 (8)	-
TGD + Cisgender	74 (11)	46 (15)	8 (7)	7 (8)	4 (4)	3 (8)	-	6 (46)	-
TGD	450 (65)	199 (64)	69 (58)	74 (80)	62 (68)	25 (64)	11 (58)	3 (23)	7 (70)
**Race / Ethnicity n (%) ***									
Asian or Pacific Islander	151 (22)	86 (28)	16 (13)	11 (12)	22 (24)	8 (21)	4 (21)	2 (15)	2 (20)
Black or African Descent	200 (29)	117 (38)	19 (16)	14 (15)	31 (34)	9 (23)	5 (26)	3 (23)	2 (20)

Hispanic or Latinx	172 (25)	106 (34)	15 (13)	11 (12)	25 (27)	7 (18)	4 (21)	2 (15)	2 (20)
Indigenous or Aboriginal	80 (11)	54 (17)	8 (7)	-	9 (10)	4 (10)	2 (11)	1 (8)	2 (20)
Multiple Races/Ethnicities	119 (17)	67 (21)	11 (10)	3 (3)	27 (30)	6 (39)	2 (11)	1 (8)	2 (20)
Not Disclosed or Unknown	103 (15)	57 (18)	13 (11)	10 (11)	8 (9)	6 (15)	6 (32)	3 (23)	-
Not Reported	409 (59)	158 (51)	85 (71)	73 (79)	41 (45)	26 (67)	10 (53)	9 (69)	7 (70)
Other	153 (22)	100 (32)	17 (14)	12 (13)	12 (13)	3 (8)	6 (32)	2 (15)	1 (10)
White	264 (38)	144 (46)	32 (27)	17 (18)	43 (47)	13 (33)	8 (42)	4 (31)	3 (30)
**Age Groups Represented n (Years; %) ***									
12 ≤ age	91 (13)	37 (12)	28 (23)	10 (11)	9 (10)	3 (8)	2 (11)	1 (8)	1 (10)
13 ≤ age ≤ 17	193 (28)	94 (30)	36 (30)	22 (24)	21 (23)	9 (23)	3 (16)	2 (15)	6 (60)
18 ≤ age ≤ 29	446 (64)	201 (64)	75 (63)	57 (62)	63 (69)	28 (72)	11 (58)	3 (23)	8 (80)
30 ≤ age ≤ 50	374 (54)	168 (54)	60 (50)	54 (59)	49 (54)	23 (59)	12 (63)	5 (38)	3 (30)
51 ≥ age	263 (38)	132 (42)	35 (29)	35 (38)	34 (38)	14 (36)	6 (32)	4 (31)	2 (20)

Notes. White: minimum value (%). Darkest shade of grey: maximum value (%). Values in between are indicated by the gradient. LGBTQ: Lesbian, Queer, Bisexual, Transgender, and Queer. Questioning: Individuals who were questioning their gender identity. TGD: Transgender and Gender-Diverse. ***:** Percentages add up to more than 100% and n adds up to more than total number of studies due to multiple age groups and/or racial/ethnic groups being represented in some studies.

Fig 2. Percentage of studies as per numeric value which included participants with a specified gender identity across all 18 chapters. LGBTQ: Lesbian, Gay, Bisexual, Transgender, and Queer. Questioning: Individuals who were questioning their gender identity. TGD: Transgender and Gender-diverse.

Temporal patterns in the gender composition of study populations

Despite the gender composition of studies published yearly since 1969 remaining stable (**Fig 3A**), there has been significant increase (P=.03) in the proportion of studies involving both TGD and cisgender individuals and a significant decrease in the proportion of studies involving study participants with an unspecified gender identity (P=.04; **Fig 3B**). The proportions TGD-only (P=.36) or cisgender-only studies (P=.82) did not change over the same time period.

Fig 3. Temporal patterns among studies cited in the WPATH SOC 8. A) Absolute number of studies published and gender identity of participants as a function of time. B) Gender identity of participants involved in studies over time expressed as percentage. Ptrend calculated for the proportion of studies involving exclusively TGD individuals, not specifying gender identity, both TGD and cisgender individuals, and exclusively cisgender individuals. TGD: Transgender and Gender-diverse. Given terms including eunuch, intersex, LGBTQ, do not provide sufficient information to categorize an individual's gender identity, they were included in the "Not Specified" group. Participants who were questioning their gender identity were included in the "TGD" group.

Study characteristics informing hormone therapy guidance

Of 1809 cited articles, 115 original studies met the inclusion criteria (**Table 2**). The median year of publication was 2018 (range: 1981 to 2022). TGD-only populations were represented in 72% of studies, with 18 to 29 years of age being the most represented age group across all studies (63%), followed by 30 to 50 years of age (58%). Race/ethnicity was not reported in 57% of studies. Of the studies that did report race/ethnicity, white participants had the highest representation (42%), followed by Black or African descent (30%), Asian or Pacific Islander (28%), and Hispanic/ Latinx (26%). The most common study design was cross-sectional (34%), with randomized controlled trials (RCTs) representing 8% of studies. Less than half of studies stratified results by sex (48%) or gender (39%), and only a minority included sex (2%) or gender (2%) as a covariate in relevant analyses (Data not shown).

Table 2. Summary of study characteristics of studies cited in chapter 12, titled "Hormone Therapy".

	All<br>n = 115 (100%)	Cross Sectional<br>n = 39 (34%)	Prospective Cohort<br>n = 29 (25%)	Retrospective Cohort<br>n = 19 (17%)	Qualitative<br>n = 4 (3%)	Mixed-Methods<br>n = 8 (7%)	Randomized Controlled Trial<br>n = 9 (8%)	Case Control<br>n = 5 (4%)	Pre-Post<br>n = 2 (2%)
**Median Year (Range)**	2018 (1981 –2022)	2017 (2000 – 2021)	2018 (1981 – 2022)	2019 (1997 – 2021)	2018 (2015 – 2020)	2014 (2008 – 2021)	2013 (2003 – 2021)	2014 (2007 – 2019)	2019.5 (2019 – 2020)
**Gender Identities n (%)**									
Cisgender	9 (8)	1 (3)	2 (7)	1 (5)	-	-	2 (22)	3 (60)	-
Intersex	-	-	-	-	-	-	-	-	-
Eunuch	-	-	-	-	-	-	-	-	-
LGBTQ	2 (2)	-	-	-	1 (25)	1 (12)	-	-	-
Not Reported	4 (3)	2 (5)	2 (7)	-	-	-	-	-	-
Questioning	8 (7)	1 (3)	5 (17)	1 (5)	-	1 (12)	-	-	-
TGD + Cisgender	9 (8)	5 (13)	2 (7)	-	-	1 (12)	-	1 (20)	-
TGD	83 (72)	30 (77)	18 (62)	17 (90)	3 (75)	5 (63)	7 (78)	1 (20)	2 (100)
**Race / Ethnicity n (%) ***									
Asian or Pacific Islander	32 (28)	14 (36)	4 (14)	5 (26)	2 (50)	2 (25)	3 (33)	1 (20)	1 (50)
Black or African Descent	35 (30)	16 (41)	5 (17)	4 (21)	2 (50)	2 (25)	4 (44)	1 (20)	1 (50)

Hispanic or Latinx	30 (26)	13 (33)	4 (14)	5 (26)	2 (50)	2 (25)	3 (33)	-	1 (50)
Indigenous or Aboriginal	17 (15)	9 (23)	3 (10)	-	1 (25)	1 (13)	2 (22)	-	1 (50)
Multiple Races/Ethnicities	19 (17)	11 (28)	2 (7)	1 (5)	3 (75)	1 (13)	-	-	1 (50)
Not Disclosed or Unknown	28 (24)	10 (26)	5 (17)	6 (32)	-	2 (29)	4 (44)	1 (20)	-
Not reported	65 (57)	19 (49)	20 (69)	13 (68)	-	5 (63)	4 (44)	3 (60)	1 (50)
Other	26 (23)	13 (33)	5 (17)	3 (16)	1 (25)	-	3 (33)	1 (20)	-
White	48 (42)	19 (49)	8 (28)	6 (32)	4 (100)	3 (38)	5 (56)	2 (40)	1 (50)
**Age Groups Represented n (Years; %) ***									
12 ≤ age	11 (10)	3 (8)	5 (13)	3 (16)	-	-	-	-	-
13 ≤ age ≤ 17	30 (26)	11 (28)	8 (21)	6 (32)	1 (25)	2 (25)	-	1 (20)	1 (50)
18 ≤ age ≤ 29	72 (63)	23 (59)	19 (49)	13 (68)	3 (75)	5 (63)	6 (67)	2 (40)	1 (50)
30 ≤ age ≤ 50	67 (58)	21 (54)	15 (38)	12 (63)	2 (50)	3 (38)	9 (100)	5 (100)	-
51 ≥ age	41 (36)	16 (41)	6 (15)	7 (37)	2 (50)	1 (13)	5 (56)	4 (80)	-

Notes. White: minimum value (%). Darkest shade of grey: maximum value (%). Values in between are indicated by the gradient. LGBTQ: Lesbian, Queer, Bisexual, Transgender, and Queer. Questioning: Individuals who were questioning their gender identity. TGD: Transgender and Gender-Diverse. ***:** Percentages add up to more than 100% and n adds up to more than total number of studies due to multiple age groups and/or racial/ethnic groups being represented in some studies.

Discussion

We sought to characterize the literature directly informing care of TGD persons. Our key findings were as follows: 1) two-thirds of studies included TGD-only populations, 2) the proportion of studies with populations that include both TGD and cisgender participants has increased over time, while the proportion of studies with study populations of unspecified gender identity has decreased over time, 3) the majority of studies included cross-sectional studies within young adults age ranges, and 4) while the majority of studies did not report the race/ethnicity of participants, white participants were most commonly represented in studies listing this demographic. While these results suggest a change towards improved inclusion of methods allowing for greater representation of a diversity of participants within health research, and thus providing an improved evidence base informing the care of TGD persons, much work remains.

Our results are consistent with previous reports also showing a shift towards more TGD-centric health research, although we were not able to determine if this shift was inclusive of all or only limited to specific gender identities. In a bibliometric analysis of peer-reviewed literature in TGD health between 1900 and 2017, an exponential increase in the number of TGD health articles was observed over the last decade (15). However, as outlined previously (16-18), more comprehensive long-term research centering a diversity of TGD health experiences across life stages is required to optimally inform care of persons of different gender identities. In particular, guidance related to gender-affirming hormone therapy is informed by the principles of hormone replacement treatment of hypogonadal individuals (19), which may or may not reflect the needs of TGD individuals.

Previous work has outlined the perils of generalizing results from one population to other populations (3, 20-22); our analysis show that in addition to a much larger absolute number of cisgender participants compared to TGD participants across all studies in all chapters, nine studies included cisgender participants exclusively, and these nine studies were all used to inform gender-affirming hormone therapy in TGD individuals.

The percentage of older adults (individuals aged ≥65 years (23) is projected to double from 12% to 22% by 2050 (24) due to declining fertility and increasing longevity (25). Although numerous studies included participants from across the lifespan, young adults were the most frequently captured age group, which may reflect a primary research focus on time periods just before or early into the gender transition process (16), thus limiting the potential for informed care of TGD individuals in the decades after medical transition or older adults initiating the gender transition process. Underrepresentation of older adults in clinical research is widespread across virtually all fields (26-32) with resulting care guidelines often relying on studies with limited older participants, potentially reducing their applicability in this age group (33-35). A recent review (36) outlined common barriers and potential solutions to increase participation of older adults in clinical research, which coupled with TGD-inclusive practices (37, 38) could lead to a greater evidence base in the health care of older adults.

It is noteworthy that cross-sectional studies were the most common type of study informing care of TGD persons and that this study type is not designed to determine causal relationships (39). Two recent reviews exploring TGD research also found cross-sectional studies to be the most common type of study (18, 40). As outlined above, there is a need for longitudinal studies examining TGD health throughout the lifespan (16, 18).

The quality of evidence from health research is partially deemed by the hierarchy of study designs, where observational studies are considered of lower quality evidence compared to RCTs, and systematic reviews and meta-analyses. As previously highlighted, increased scientific rigor and reach of TGD health research is required to ultimately inform evidence-based prevention and care for this underserved population (41).

It is encouraging that our observations show an increase in the proportion of studies including both TGD and cisgender participants, in parallel to a decrease in the proportion of studies with study populations of unspecified gender identity. These observations are likely due to a myriad of factors, including changes in research methods and approaches to data collection and policy (10) as well as increased availability of educational resources, guidelines, and frameworks (42-45). However, in studies where both TGD and cisgender participants were included, for every one TGD individual there were two cisgender individuals, and the median number of cisgender participants was approximately triple that of the median number of TGD participants included across all studies. Previous studies have highlighted the importance of the participation to prevalence ratio (PPR), a measure of the representation of a particular group in the study population relative to the prevalence of the condition of interest in the same group (46). While we did not stratify studies according to condition, study populations including both TGD and cisgender participants should be representative of the prevalence of the condition under examination. The low proportions of studies incorporating sex and gender identity in analysis of results are in keeping with previous studies (17, 18, 47), highlighting that both inclusion and disaggregation of sex and gender is required to support sex- and gender-based analyses of not only TGD health research, but all health research.

This study has limitations. First, only studies referenced in the WPATH SOC 8 were included, rather than the entire body of literature informing TGD healthcare. However, WPATH is an international and multidisciplinary professional association whose mission is to promote evidence-based care of TGD persons; one of the main functions of WPATH is to promote the highest standards of health care for TGD persons through the Standards of Care (12) “based on the best available science”. Next, studies where the population of interest assessing caregivers, providers, siblings, partners, or children of TGD individuals were excluded, thus centering the focus on only TGD individuals, which does not capture issues in the broader environment or family systems that play important roles in health outcomes (16). However, as the aim of this work as to specifically examine TGD representation in health research informing the care of TGD persons, inclusion of these other groups would not have contributed to this goal. Finally, WPATH SOC 8 was published in 2022, and as such new scientific evidence published since its release was not captured in this study. In February 2024, a literature search on Pubmed.gov using the keyword “transgender” and “2023” revealed 134 RCTs, systematic reviews and meta-analyses, which may reflect an even greater evidence base for the care of TGD individuals.

Conclusions

This review was aimed to characterize the literature informing the care of TGD persons as outlined in the WPATH SOC version 8 guidelines. While it is encouraging to observe that TGD participant-only studies represent the majority of this literature, the quality of evidence is an area of concern and greater attention to inclusion of a diversity of TGD individuals across the lifespan and especially as TGD individuals age, as well as

community engagement in all steps of the research process, is warranted (16-18, 37, 40). In this era of precision health (48), the growing number of individuals who identify as TGD (49-51) coupled with the increasingly recognized health inequities within this understudied and underserved population (2, 52) underscores the need for urgent action.

Acknowledgements

N/A

Supporting Information

S1 File. Data for individual chapters, excluding Chapter 12.

References

1. Caughey AB, Krist AH, Wolff TA, Barry MJ, Henderson JT, Owens DK, et al. USPSTF Approach to Addressing Sex and Gender When Making Recommendations for Clinical Preventive Services. JAMA. 2021;326(19):1953-61.
2. Jackson SS, Brown J, Pfeiffer RM, Shrewsbury D, O'Callaghan S, Berner AM, et al. Analysis of Mortality Among Transgender and Gender Diverse Adults in England. JAMA Network Open. 2023;6(1):e2253687-e.
3. Bibbins-Domingo K, Helman A, Dzau VJ. The Imperative for Diversity and Inclusion in Clinical Trials and Health Research Participation. Jama. 2022;327(23):2283-4.
4. National Academies of Sciences E, Medicine, Division of B, Social S, Education, Committee on National S, et al. The National Academies Collection: Reports funded by National Institutes of Health. In: Becker T, Chin M, Bates N, editors. Measuring Sex, Gender Identity, and Sexual Orientation. Washington (DC): National Academies Press (US) Copyright 2022 by the National Academy of Sciences. All rights reserved.; 2022.
5. Streed CG, Jr., Perlson JE, Abrams MP, Lett E. On, With, By-Advancing Transgender Health Research and Clinical Practice. Health Equity. 2023;7(1):161-5.
6. National Academies of Sciences E, Medicine, Policy, Global A, Committee on Women in Science E, Medicine, et al. The National Academies Collection: Reports funded by National Institutes of Health. In: Bibbins-Domingo K, Helman A, editors. Improving Representation in Clinical Trials and Research: Building Research Equity for Women and Underrepresented Groups. Washington (DC): National Academies Press (US) Copyright 2022 by the National Academy of Sciences. All rights reserved.; 2022.
7. Health NIo. NIH Policy on Sex as a Biological Variable 2015 [Available from: https://orwh.od.nih.gov/sex-gender/orwh-mission-area-sex-gender-in-research/nih-policy-on-sex-as-biological-variable.
8. Health NIo. Amendment: NIH Policy and Guidelines on the Inclusion of Women and Minorities as Subjects in Clinical Research 2017 [Available from: https://grants.nih.gov/grants/guide/notice-files/NOT-OD-18-014.html.
9. Health NIo. NIH Revitalization Act of 1993. Public Law. 1993:103-43.
10. ClinicalTrials.gov. Protocol Registration Data Element Definitions for Interventional and Observational Studies 2020 [Available from: https://prsinfo.clinicaltrials.gov/definitions.html.
11. Burton A, Mohan S, Puplampu-Dove Y, Tse T. Characterizing Gender Eligibility Descriptions for Clinical Trials Registered on ClinicalTrials.gov. Jama. 2023;330(20):2019-21.
12. Coleman E, Radix AE, Bouman WP, Brown GR, de Vries ALC, Deutsch MB, et al. Standards of Care for the Health of Transgender and Gender Diverse People, Version 8. Int J Transgend Health. 2022;23(Suppl 1):S1-s259.
13. Google. Google Translate 2006 [Available from: https://translate.google.ca.
14. Armitage P. Tests for Linear Trends in Proportions and Frequencies. Biometrics. 1955;11(3):375-86.
15. Sweileh WM. Bibliometric analysis of peer-reviewed literature in transgender health (1900 - 2017). BMC Int Health Hum Rights. 2018;18(1):16.

16. Veale JF, Deutsch MB, Devor AH, Kuper LE, Motmans J, Radix AE, et al. Setting a research agenda in trans health: An expert assessment of priorities and issues by trans and nonbinary researchers. International Journal of Transgender Health. 2022;23(4):392-408.
17. Scheim A, Kacholia V, Logie C, Chakrapani V, Ranade K, Gupta S. Health of transgender men in low-income and middle-income countries: a scoping review. BMJ Glob Health. 2020;5(11).
18. Marshall Z, Welch V, Minichiello A, Swab M, Brunger F, Kaposy C. Documenting Research with Transgender, Nonbinary, and Other Gender Diverse (Trans) Individuals and Communities: Introducing the Global Trans Research Evidence Map. Transgend Health. 2019;4(1):68-80.
19. Hembree WC, Cohen-Kettenis PT, Gooren L, Hannema SE, Meyer WJ, Murad MH, et al. Endocrine Treatment of Gender-Dysphoric/Gender-Incongruent Persons: An Endocrine Society* Clinical Practice Guideline. The Journal of Clinical Endocrinology & Metabolism. 2017;102(11):3869-903.
20. Levin RI. The puzzle of aspirin and sex. N Engl J Med. 2005;352(13):1366-8.
21. Desir FA, Lesko CR, Moore RD, Horberg MA, Wong C, Crane HM, et al. One Size Fits (n)One: The Influence of Sex, Age, and Sexual Human Immunodeficiency Virus (HIV) Acquisition Risk on Racial/Ethnic Disparities in the HIV Care Continuum in the United States. Clin Infect Dis. 2019;68(5):795-802.
22. Meadows TA, Bhatt DL, Cannon CP, Gersh BJ, Röther J, Goto S, et al. Ethnic differences in cardiovascular risks and mortality in atherothrombotic disease: insights from the Reduction of Atherothrombosis for Continued Health (REACH) registry. Mayo Clin Proc. 2011;86(10):960-7.
23. Singh S, Bajorek B. Defining 'elderly' in clinical practice guidelines for pharmacotherapy. Pharm Pract (Granada). 2014;12(4):489.
24. Safer JD, Coleman E, Feldman J, Garofalo R, Hembree W, Radix A, et al. Barriers to healthcare for transgender individuals. Current Opinion in Endocrinology, Diabetes and Obesity. 2016;23(2).
25. UN. Ageing [Available from: https://www.un.org/en/global-issues/ageing#:~:text=While%20declining%20fertility%20and%20increasing,in%20some%20countries%20and%20regions.
26. Banzi R, Camaioni P, Tettamanti M, Bertele V, Lucca U. Older patients are still under-represented in clinical trials of Alzheimer's disease. Alzheimers Res Ther. 2016;8:32.
27. Dodd KS, Saczynski JS, Zhao Y, Goldberg RJ, Gurwitz JH. Exclusion of older adults and women from recent trials of acute coronary syndromes. J Am Geriatr Soc. 2011;59(3):506-11.
28. Hutchins LF, Unger JM, Crowley JJ, Coltman CA, Jr., Albain KS. Underrepresentation of patients 65 years of age or older in cancer-treatment trials. N Engl J Med. 1999;341(27):2061-7.
29. Kanapuru B, Singh H, Kwitkowski V, Blumenthal G, Farrell AT, Pazdur R. Older adults in hematologic malignancy trials: Representation, barriers to participation and strategies for addressing underrepresentation. Blood Rev. 2020;43:100670.
30. Konrat C, Boutron I, Trinquart L, Auleley GR, Ricordeau P, Ravaud P. Underrepresentation of elderly people in randomised controlled trials. The example of trials of 4 widely prescribed drugs. PLoS One. 2012;7(3):e33559.
31. Palmowski A, Buttgereit T, Palmowski Y, Nielsen SM, Boers M, Christensen R, et al. Applicability of trials in rheumatoid arthritis and osteoarthritis: A systematic review and meta-

analysis of trial populations showing adequate proportion of women, but underrepresentation of elderly people. Semin Arthritis Rheum. 2019;48(6):983-9.
32. Gurwitz JH, Col NF, Avorn J. The exclusion of the elderly and women from clinical trials in acute myocardial infarction. Jama. 1992;268(11):1417-22.
33. Sardar MR, Badri M, Prince CT, Seltzer J, Kowey PR. Underrepresentation of women, elderly patients, and racial minorities in the randomized trials used for cardiovascular guidelines. JAMA Intern Med. 2014;174(11):1868-70.
34. O'Hare AM, Kaufman JS, Covinsky KE, Landefeld CS, McFarland LV, Larson EB. Current guidelines for using angiotensin-converting enzyme inhibitors and angiotensin II-receptor antagonists in chronic kidney disease: is the evidence base relevant to older adults? Ann Intern Med. 2009;150(10):717-24.
35. Lugtenberg M, Burgers JS, Clancy C, Westert GP, Schneider EC. Current guidelines have limited applicability to patients with comorbid conditions: a systematic analysis of evidence-based guidelines. PLoS One. 2011;6(10):e25987.
36. Forsat ND, Palmowski A, Palmowski Y, Boers M, Buttgereit F. Recruitment and Retention of Older People in Clinical Research: A Systematic Literature Review. J Am Geriatr Soc. 2020;68(12):2955-63.
37. Rytz CL, Beach LB, Saad N, Dumanski SM, Collister D, Newbert AM, et al. Improving the inclusion of transgender and nonbinary individuals in the planning, completion, and mobilization of cardiovascular research. American Journal of Physiology-Heart and Circulatory Physiology. 2023;324(3):H366-H72.
38. Owen-Smith AA, Woodyatt C, Sineath RC, Hunkeler EM, Barnwell T, Graham A, et al. Perceptions of Barriers to and Facilitators of Participation in Health Research Among Transgender People. Transgend Health. 2016;1(1):187-96.
39. Wang X, Cheng Z. Cross-Sectional Studies: Strengths, Weaknesses, and Recommendations. Chest. 2020;158(1s):S65-s71.
40. Scheim AI, Rich AJ, Zubizarreta D, Malik M, Baker KE, Restar AJ, et al. Health status of transgender people globally: A systematic review of research on disease burden and correlates. PLoS One. 2024;19(3):e0299373.
41. Reisner SL, Deutsch MB, Bhasin S, Bockting W, Brown GR, Feldman J, et al. Advancing methods for US transgender health research. Curr Opin Endocrinol Diabetes Obes. 2016;23(2):198-207.
42. Health NIo. The Sexual & Gender Minority Research Office 2015 [Available from: https://dpcpsi.nih.gov/sgmro.
43. Heidari S, Babor TF, De Castro P, Tort S, Curno M. Sex and Gender Equity in Research: rationale for the SAGER guidelines and recommended use. Research Integrity and Peer Review. 2016;1(1):2.
44. Clayton JA, Tannenbaum C. Reporting Sex, Gender, or Both in Clinical Research? Jama. 2016;316(18):1863-4.
45. Ghorbanian A, Aiello B, Staples J. Under-Representation of Transgender Identities in Research: The Limitations of Traditional Quantitative Survey Data. Transgend Health. 2022;7(3):261-9.

46. Poon R, Khanijow K, Umarjee S, Fadiran E, Yu M, Zhang L, et al. Participation of women and sex analyses in late-phase clinical trials of new molecular entity drugs and biologics approved by the FDA in 2007-2009. J Womens Health (Larchmt). 2013;22(7):604-16.
47. Gulamhusein N, Turino Miranda K, Dumanski SM, González Bedat MC, Ulasi I, Conjeevaram A, et al. Sex- and Gender-Based Reporting in Antihypertensive Medication Literature Informing Hypertension Guidelines. Journal of the American Heart Association. 2024;0(0):e030613.
48. Hudson K, Lifton, R., Patrick-Lake, B., Burchard. E.G., Coles, T., Collins. R., Conrad, A., Denny, J., Desmond-Hellmann, S., Dishman, E., Califf, R., DeSalvo, K., Handelsman, J., Jenkins, G.L., Giusti, K., Kathiresan, S., Kheterpal, S., Kumanyika, S., Manson, S.M., O'Rourke, P.P., Platt, R., Shendure, J., Siegel, S., O'Leary, T., Raunch, T.M. The Precision Medicine Initiative Cohort Program – Building a Research Foundation for 21st Century Medicine 2015 [Available from: https://acd.od.nih.gov/documents/reports/DRAFT-PMI-WG-Report-9-11-2015-508.pdf.
49. Canada is the first country to provide census data on transgender and non-binary people: Statistics Canada; 2021 [Available from: https://www150.statcan.gc.ca/n1/daily-quotidien/220427/dq220427b-eng.htm.
50. Meerwijk EL, Sevelius JM. Transgender Population Size in the United States: a Meta-Regression of Population-Based Probability Samples. Am J Public Health. 2017;107(2):e1-e8.
51. Collin L, Reisner SL, Tangpricha V, Goodman M. Prevalence of Transgender Depends on the "Case" Definition: A Systematic Review. J Sex Med. 2016;13(4):613-26.
52. Fredriksen Goldsen KI, Romanelli M, Hoy-Ellis CP, Jung H. Health, economic and social disparities among transgender women, transgender men and transgender nonbinary adults: Results from a population-based study. Prev Med. 2022;156:106988.

Fig1

Click here to access/download;Figure;Fig1.tif

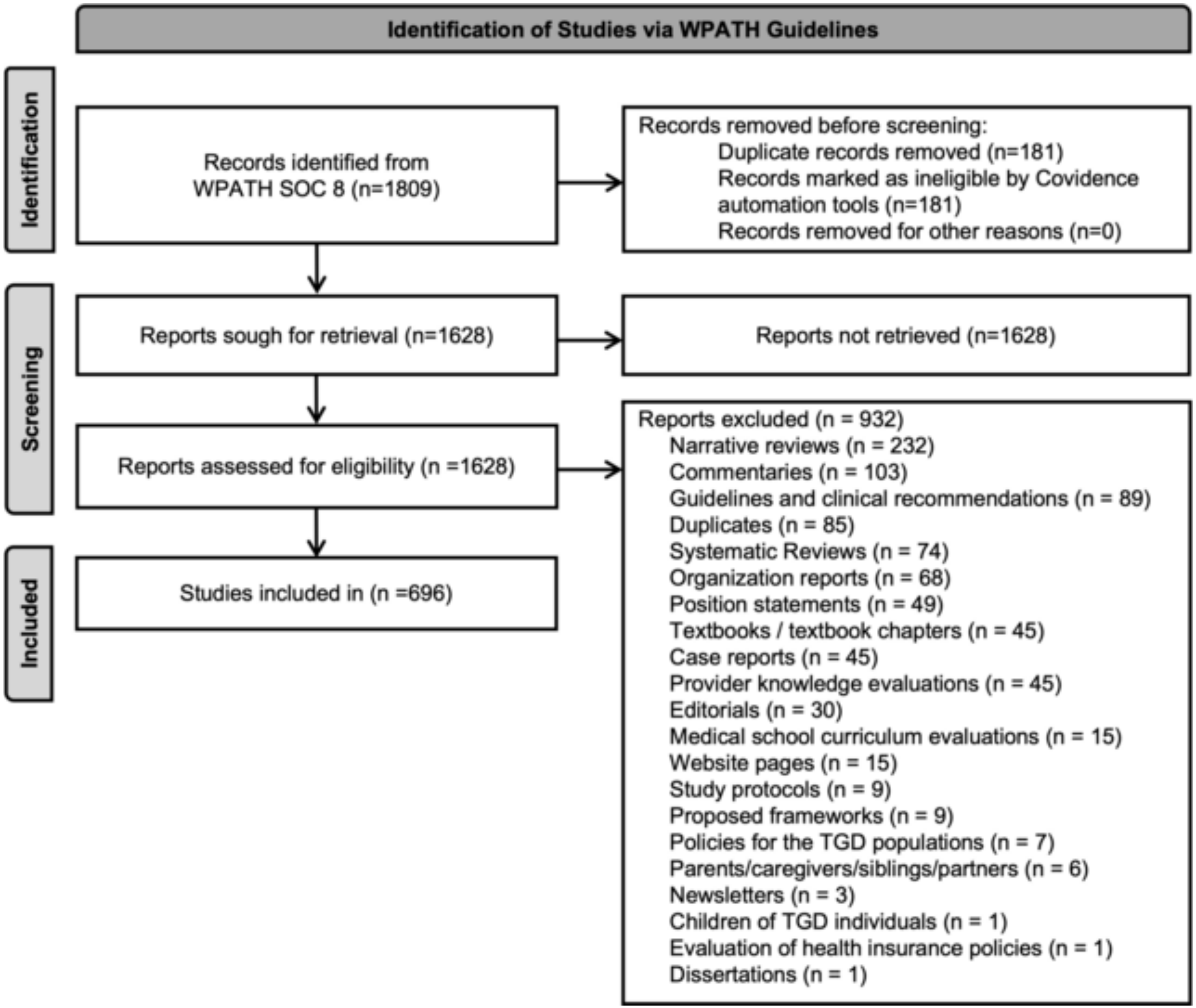

Fig2

Click here to access/download;Figure;Fig2.tif

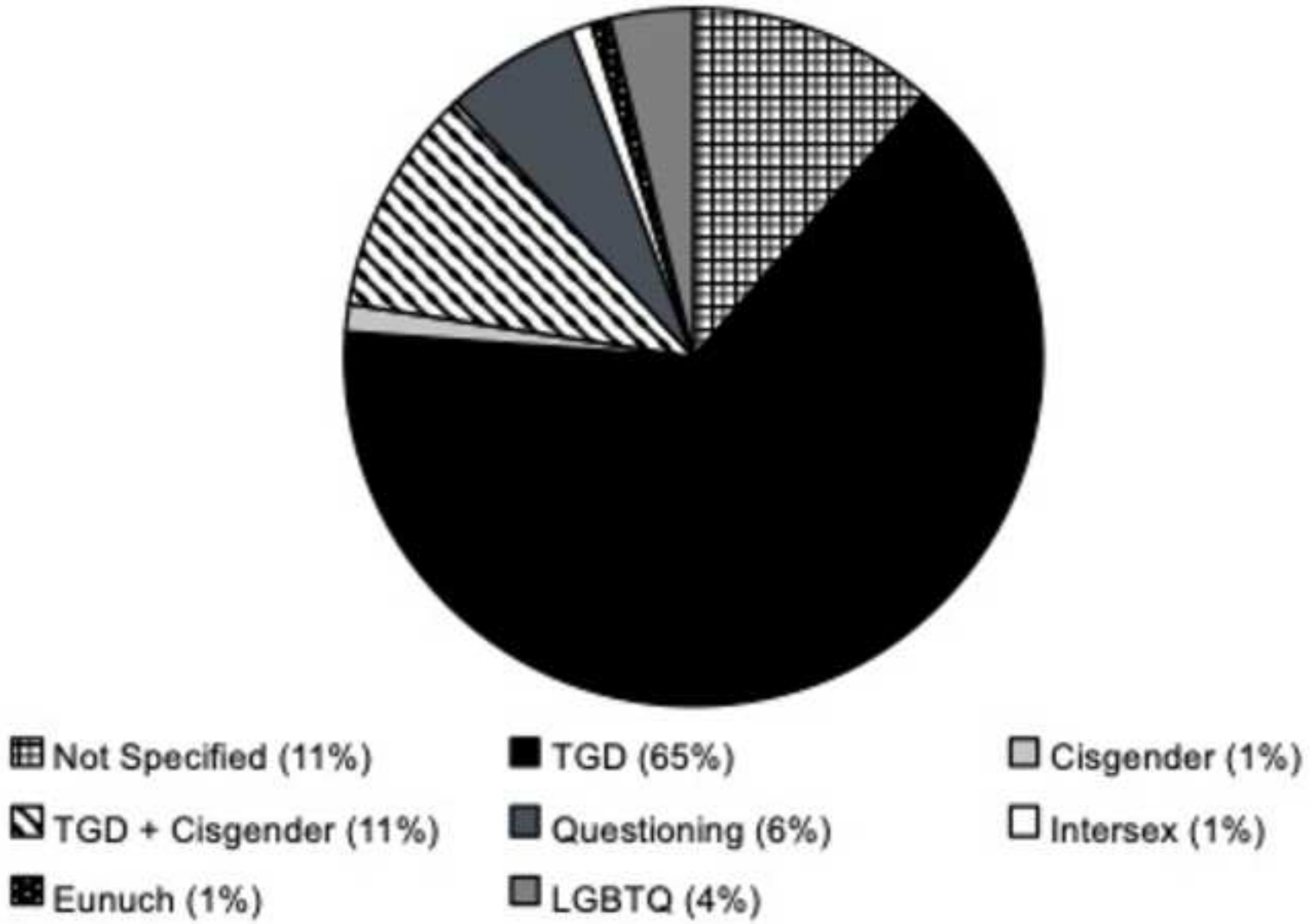

Fig3

Click here to access/download;Figure;Fig3.tif

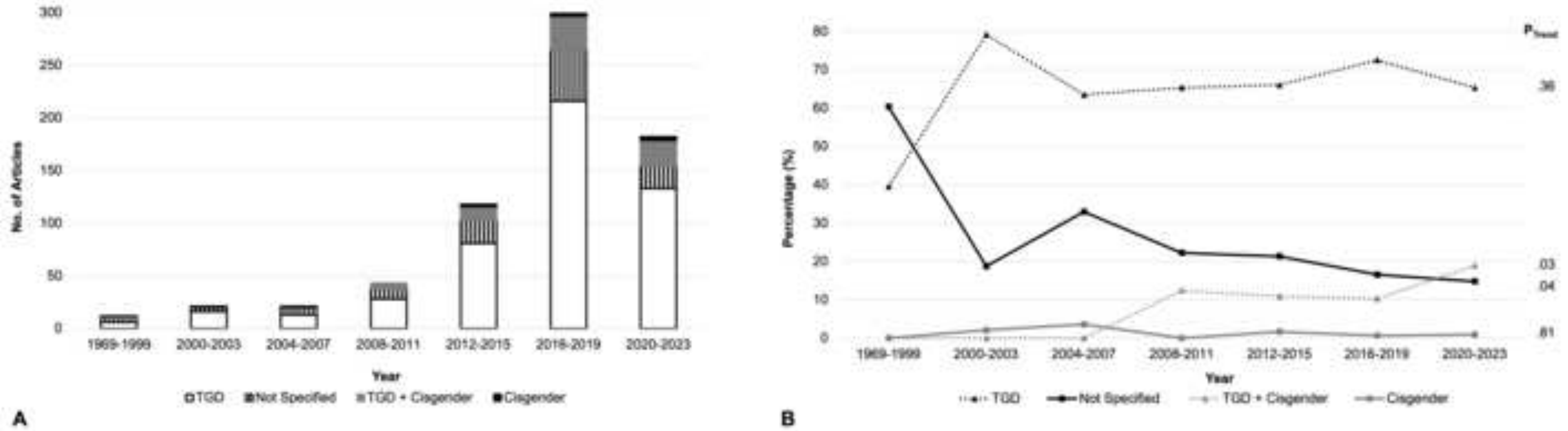

S1 File. Data for individual chapters, excluding Chapter 12.

Click here to access/download
Supporting Information
S1 File.docx
