# Supplementary material for: Characterization of the literature informing health care of transgender and gender-diverse persons: A bibliometric analysis
Source: PLoS One. 2024 Oct 4;19(10):e0309169. doi: 10.1371/journal.pone.0309169 (PMC11452042; doi:10.1371/journal.pone.0309169)
Supplement: Attachment — Submitted filename: Response to Reviewers.docx [file pone.0309169.s004.docx]

July 8, 2024

Emily Chenette, PhD 
Editor-in-Chief 
PLOS ONE

**Manuscript ID#: *PONE-D-24-13832.R1***

**Title:** Characterization of the literature informing health care of transgender and gender-diverse persons: A bibliometric analysis

Thank you for allowing us the opportunity to resubmit our manuscript, based on the thorough review and thoughtful comments provided by the Reviewers. We feel that we have been able to satisfactorily address all the comments raised, which has further enhanced the quality of the manuscript. We have provided an itemized summary of the changes made to the paper below with the Reviewers’ comments provided in bold, followed by our responses. We have provided a tracked changed version of the manuscript, as well as a version without track changes.

**Editor:**

**C1. I share the reviewer’s concerns about Figure 2 in your manuscript. What information does this figure convey that is not already presented in Table 1? If you want to retain the figure, I suggest making it more apparent what additional information is being presented.**

R1. We thank the Editor for this comment. Figure 2 has been removed from the manuscript.

**C2. Please ensure that your manuscript meets PLOS ONE's style requirements, including those for file naming. The PLOS ONE style templates can be found at**

**https://journals.plos.org/plosone/s/file?id=wjVg/PLOSOne_formatting_sample_main_body.pdf and**

**https://journals.plos.org/plosone/s/file?id=ba62/PLOSOne_formatting_sample_title_authors_affiliations.pdf**

R2. We thank the Editor for this comment. The manuscript has now been formatted to meets PLOS One's style requirements.

**C3. We note that the grant information you provided in the ‘Funding Information’ and ‘Financial Disclosure’ sections do not match.**

**When you resubmit, please ensure that you provide the correct grant numbers for the awards you received for your study in the ‘Funding Information’ section.**

R3. Thank you for this comment. This study was unfunded, and the ‘Funding Information’ section has been updated accordingly.

**C4. When completing the data availability statement of the submission form, you indicated that you will make your data available on acceptance. We strongly recommend all authors decide on a data sharing plan before acceptance, as the process can be lengthy and hold up publication timelines. Please note that, though access restrictions are acceptable now, your entire data will need to be made freely accessible if your manuscript is accepted for publication. This policy applies to all data except where public deposition would breach compliance with the protocol approved by your research ethics board. If you are unable to adhere to our open data policy, please kindly revise your statement to explain your reasoning and we will seek the editor's input on an exemption. Please be assured that, once you have provided your new statement, the assessment of your exemption will not hold up the peer review process.**

R4. We thank the Editor for this comment. We have submitted a supplementary file, which includes our entire dataset to generate all figures and tables found in our manuscript. We have also listed this file under the “Supporting Information” header one page 22, line 317:

“S2 File. Studies and study characteristics included in bibliometric analysis.”

**C5. We note that you have included the phrase “data not shown” in your manuscript. Unfortunately, this does not meet our data sharing requirements. PLOS does not permit references to inaccessible data. We require that authors provide all relevant data within the paper, Supporting Information files, or in an acceptable, public repository. Please add a citation to support this phrase or upload the data that corresponds with these findings to a stable repository (such as Figshare or Dryad) and provide and URLs, DOIs, or accession numbers that may be used to access these data. Or, if the data are not a core part of the research being presented in your study, we ask that you remove the phrase that refers to these data.**

R5. We thank the Editor for this comment. The phrase “data not shown” has been removed. We have now indicated that these results are reported in Table 1 and 2, as well as the supplementary file.

**C6. Please review your reference list to ensure that it is complete and correct. If you have cited papers that have been retracted, please include the rationale for doing so in the manuscript text, or remove these references and replace them with relevant current references. Any changes to the reference list should be mentioned in the rebuttal letter that accompanies your revised manuscript. If you need to cite a retracted article, indicate the article’s retracted status in the References list and also include a citation and full reference for the retraction notice.**

We thank the Editor for the comment. The following references have been updated:

Line 327, citation #4: National Academies of Sciences, Engineering and Medicine. Measuring Sex, Gender Identity, and Sexual Orientation. Washington (DC): National Academies Press (US). 2022.

Line 333, citation #6: National Academies of Sciences, Engineering and Medicine. Improving Representation in Clinical Trials and Research: Building Research Equity for Women and Underrepresented Groups. Washington (DC): National Academies Press (US). 2022.

Line 337, citation #7: National Institutes of Health Research: Office of Research on Women’s Health. NIH Policy on Sex as a Biological Variable 2015 [Available from: https://orwh.od.nih.gov/sex-gender/orwh-mission-area-sex-gender-in-research/nih-policy-on-sex-as-biological-variable].

Line 341, citation #8: National Institutes of Health Research. Amendment: NIH Policy and Guidelines on the Inclusion of Women and Minorities as Subjects in Clinical Research 2017 [Available from: https://grants.nih.gov/grants/guide/notice-files/NOT-OD-18-014.html].

Line 344, citation #9: National Institutes of Health Research. NIH Revitalization Act of 1993. Public Law. 1993:103-43.

Line 395, citation #28: United Nations. Ageing [Available from: https://www.un.org/en/global-issues/ageing#:~:text=While%20declining%20fertility%20and%20increasing,in%20some%20countries%20and%20regions].

Line 447, citation #45: National Institutes of Health Research. The Sexual & Gender Minority Research Office 2015 [Available from: https://dpcpsi.nih.gov/sgmro].

Line 498, citation #63: Hudson K., Lifton, R., Patrick-Lake, B., Burchard. E.G., Coles, T., Collins. R., et al. The Precision Medicine Initiative Cohort Program – Building a Research Foundation for 21st Century Medicine 2015 [Available from: https://acd.od.nih.gov/documents/reports/DRAFT-PMI-WG-Report-9-11-2015-508.pdf].

The following references have been added to address the Reviewers’ constructive comments:

Line 356, citation #14: Jackson JL, Kuriyama A, Anton A, Choi A, Fournier J-P, Geier A-K, et al. The Accuracy of Google Translate for Abstracting Data From Non–English-Language Trials for Systematic Reviews. Annals of Internal Medicine. 2019;171(9):677-9.

Line 359, citation #15: World Health Organization. International classification of functioning, disability and health : ICF. Geneva: World Health Organization; 2001.

Line 361, citation #16: Chapman R. Defining neurodiversity for research and practice. 2020. p. 218-20.

Line 465, citation #51: Canadian Institute of Health Research. How to integrate sex and gender into research 2019 [Available from: https://cihr-irsc.gc.ca/e/50836.html].

Line 467, citation #52: National Institutes of Health Research: Center for Scientific Review. Premise, Rigor, Sex as a Biological Variable 2020 [Available from: https://public.csr.nih.gov/FAQs/ReviewersFAQs/PremiseRigorSexBiologicalVariable#7].

Line 470, citation #53: European Commission. Gender in EU research and innovation 2023 [Available from: https://rea.ec.europa.eu/gender-eu-research-and-innovation_en].

Line 472, citation #54: Medical Science Sex and Gender Equity. About MESSAGE 2023 [Available from: https://www.messageproject.co.uk/about/].

Line 474, citation #55: World Professional Association of Transgender Health. Methodology for the Development of SOC8 2022 [Available from: https://www.wpath.org/soc8/Methodology].

Line 476, citation #56: Murad MH. Clinical Practice Guidelines: A Primer on Development and Dissemination. Mayo Clin Proc. 2017;92(3):423-33.

Line 478, citation #57: Hassan R, Riehl-Tonn VJ, Dumanski SM, Lyons KJ, Ahmed SB. Female sex-specific and -predominant cardiovascular risk factors and heart failure practice guidelines. Am Heart J. 2022;247:63-7.

Line 481, citation #58: Fahmawi S, Schinke C, Thanendrarajan S, Zangari M, Shaughnessy JD, Jr., Zhan F, et al. Under-representation of black patients with multiple myeloma in studies supporting International Myeloma Working Group guidelines. J Cancer Policy. 2023;37:100433.

Line 485, citation #59: Nazer L, Abusara A, Aloran B, Szakmany T, Nabulsi H, Petushkov A, et al. Patient diversity and author representation in clinical studies supporting the Surviving Sepsis Campaign guidelines for management of sepsis and septic shock 2021: a systematic review of citations. BMC Infectious Diseases. 2023;23(1):751.

Line 489, citation #60: Lubarsky R, Ambinder D, Barnett J, Choudhury M, Saji A, Fishman AI, et al. Prostate Cancer: Under-representation of African American Men in Research Studies Used in the Latest NCCN Guidelines. Urology. 2023;180:28-34.

Line 492, citation #61: Zuberi SA, Burdine L, Dong J, Feuerstein JD. Representation of Racial Minorities in the United States Colonoscopy Surveillance Interval Guidelines. J Clin Gastroenterol. 2023.

Line 495, citation #62: Williamson TJ, Battaglia PJ, Gliedt JA, Spector AL, Williams JS. Trials Informing Back Pain Guidelines Underreport Key Sociodemographic Data. J Health Care Poor Underserved. 2023;34(1):357-76.

**Reviewer #1: 

I really appreciate this unbiased analysis of the literature from which WPATH have drawn their latest standards of care. I have a number of suggested revisions to maximise its utility to the published literature. Some of these are minor in nature in a described in comments in the PDF attached.**

**PDF Comments:**

**C1. Characterize is a broad term. Though the abstract is aiming to be brief, understanding whether aims were around quality/direction etc would the reader.**

R1. We thank the Reviewer for this comment. As we aimed to describe the literature (e.g., participant demographics, study design) cited in WPATH SOC 8, we changed the wording from “characterize” to “describe” in the abstract on line 29 of page 2:

“Thus, we aimed to describe the literature informing care provision of TGD individuals.”

**C2. This conclusion links less well to the results, as number of longitudinal studies was not specified. That would be helpful to know in the results to link this, and whether this changed over time.**

R2. Thank you for this comment. To determine the proportion of longitudinal studies cited in WPATH SOC 8, we have combined all prospective and retrospective studies, randomized controlled trials, case control and pre-post studies. The abstract has been updated to include this result on lines 39 to 40 of page 2:

“Almost half of studies (45%) were cross-sectional, and approximately a third were longitudinal in nature (37%).”

**C3. Was quality evaluation a factor? "Exploring" hormone therapy guidelines seems vague - especially if only looking at primary studies. Could more detail be provided here? As it’s such a topic of huge interest right now. I think specifying you comment on hierarchy of studies but not full analysis if their rigour e.g. matching/blinding etc.**

R3. We thank the Reviewer for this comment. We aimed to describe the literature informing TGD health care rather than evaluate the quality of studies cited. Moreover, we repeated our analysis of chapter 12, titled *Hormone Therapy*, given the recent heightened focus on this area of TGD health care. As such, we have changed the word “exploring” to “describe” and updated the objective of this study on page 5, lines 82 to 86:

“Our primary objective was to describe the participant demographics and study design of the literature cited in WPATH SOC 8 informing the care of TGD persons. Our secondary objective was to specifically describe the literature informing gender-affirming hormone therapy guidelines, given recent heightened focus on this area of TGD healthcare.”

**C4. I am concerned about the quality this might provide. Is it possible to verify with a native speaker?**

R4. We thank the Reviewer for this comment. We have updated lines 99 to 101 on page 6 to include a reference that details the accuracy of Google Translate:

“Articles in languages other than English were translated to English using Google Translate (1), which has been previously validated in systematic reviews (2).”

**C5. Given known high proportion of individuals classing themselves as having a disability, and also the high overlap with neurodivergent traits, it would be very helpful to include where this was specified.**

R5. We thank the Reviewer for this comment. We have updated the methods on page 6, lines 109 to 110:

“Data was also extracted on whether studies reported disability and neurodivergent status of participants (3, 4).”

Data on whether studies report disability and neurodivergent status, can now be found in Table 1, Table 2, in the Supplementary File and on page 7, lines 141-142:

“A minority of studies included disability (22%) or neurodivergent (5%) status of participants.”

and for the “Hormone Therapy” chapter on page 13, lines 197-198:

“Approximately a third of studies reported disability status (32%), and 6% reported neurodiversity status”

**Table 1. Summary of study characteristics of studies across all 18 chapters.**

|  | All  n = 696 (100%) | Cross Sectional  n = 312 (45%) | Prospective Cohort  n = 120 (17%) | Retrospective Cohort  n = 92 (14%) | Qualitative  n = 91 (13%) | Mixed-Methods  n = 39 (5%) | Randomized Controlled Trial  n = 19 (3%) | Case Control  n = 13 (2%) | Pre-Post  n = 10 (1%) |
| --- | --- | --- | --- | --- | --- | --- | --- | --- | --- |
| **Median Year**  **(Range)** | 2018  (1969 –2023) | 2018  (1999 – 2023) | 2017  (1969 – 2022) | 2018  (1997 – 2022) | 2018  (2008 – 2023) | 2016  (2000 – 2022) | 2019  (2003 – 2021) | 2018  (2006 – 2021) | 2016  (1997 – 2020) |
| **Gender Identities**  **n (%)** |  | | | | | | | | |
| Cisgender | 9 (1) | 1 (<1) | 2 (2) | 1 (1) | - | - | 2 (11) | 3 (23) | - |
| Eunuch | 6 (1) | 4 (1) | - | - | 1 (1) | 1 (3) | - | - | - |
| Intersex | 7 (1) | 2 (<1) | 1 (<1) | - | 2 (2) | 2 (5) | - | - | - |
| LGBTQ | 27 (4) | 12 (4) | - | 1 (1) | 9 (10) | 4 (10) | - | - | 1 (10) |
| Not Reported | 80 (11) | 32 (10) | 22 (18) | 7 (8) | 10 (11) | 2 (5) | 5 (26) | - | 2 (20) |
| Questioning | 43 (6) | 16 (5) | 18 (15) | 2 (2) | 3 (3) | 2 (5) | 1 (5) | 1 (8) | - |
| TGD + Cisgender | 74 (11) | 46 (15) | 8 (7) | 7 (8) | 4 (4) | 3 (8) | - | 6 (46) | - |
| TGD | 450 (65) | 199 (64) | 69 (58) | 74 (80) | 62 (68) | 25 (64) | 11 (58) | 3 (23) | 7 (70) |
| **Race / Ethnicity**  **n (%) *** |  | | | | | | | | |
| Asian or Pacific Islander | 151 (22) | 86 (28) | 16 (13) | 11 (12) | 22 (24) | 8 (21) | 4 (21) | 2 (15) | 2 (20) |
| Black or African Descent | 200 (29) | 117 (38) | 19 (16) | 14 (15) | 31 (34) | 9 (23) | 5 (26) | 3 (23) | 2 (20) |
| Hispanic or Latinx | 172 (25) | 106 (34) | 15 (13) | 11 (12) | 25 (27) | 7 (18) | 4 (21) | 2 (15) | 2 (20) |
| Indigenous or Aboriginal | 80 (11) | 54 (17) | 8 (7) | - | 9 (10) | 4 (10) | 2 (11) | 1 (8) | 2 (20) |
| Multiple Races/Ethnicities | 119 (17) | 67 (21) | 11 (10) | 3 (3) | 27 (30) | 6 (39) | 2 (11) | 1 (8) | 2 (20) |
| Not Disclosed or Unknown | 103 (15) | 57 (18) | 13 (11) | 10 (11) | 8 (9) | 6 (15) | 6 (32) | 3 (23) | - |
| Not Reported | 409 (59) | 158 (51) | 85 (71) | 73 (79) | 41 (45) | 26 (67) | 10 (53) | 9 (69) | 7 (70) |
| Other | 153 (22) | 100 (32) | 17 (14) | 12 (13) | 12 (13) | 3 (8) | 6 (32) | 2 (15) | 1 (10) |
| White | 264 (38) | 144 (46) | 32 (27) | 17 (18) | 43 (47) | 13 (33) | 8 (42) | 4 (31) | 3 (30) |
| **Age Groups Represented**  **n (Years; %) *** |  | | | | | | | | |
| 12 ≤ age | 91 (13) | 37 (12) | 28 (23) | 10 (11) | 9 (10) | 3 (8) | 2 (11) | 1 (8) | 1 (10) |
| 13 ≤ age ≤ 17 | 193 (28) | 94 (30) | 36 (30) | 22 (24) | 21 (23) | 9 (23) | 3 (16) | 2 (15) | 6 (60) |
| 18 ≤ age ≤ 29 | 446 (64) | 201 (64) | 75 (63) | 57 (62) | 63 (69) | 28 (72) | 11 (58) | 3 (23) | 8 (80) |
| 30 ≤ age ≤ 50 | 374 (54) | 168 (54) | 60 (50) | 54 (59) | 49 (54) | 23 (59) | 12 (63) | 5 (38) | 3 (30) |
| 51 ≥ age | 263 (38) | 132 (42) | 35 (29) | 35 (38) | 34 (38) | 14 (36) | 6 (32) | 4 (31) | 2 (20) |
| **Stratification by Gender? n (%)** |  |  |  |  |  |  |  |  |  |
| Yes | 195 (28) | 110 (35) | 32 (27) | 23 (25) | 4 (4) | 9 (23) | 4 (21) | 9 (69) | 3 (30) |
| **Gender Included as a Covariate in Analysis? n (%)** |  |  |  |  |  |  |  |  |  |
| Yes | 24 (3) | 13 (4) | 8 (7) | - | 1 (1) | - | 1 (5) | 1 (8) | - |
| **Stratification by Sex? n (%)** |  |  |  |  |  |  |  |  |  |
| Yes | 224 (32) | 128 (41) | 40 (33) | 22 (24) | 6 (7) | 10 (26) | 5 (26) | 7 (54) | 5 (50) |
| **Sex Included as**  **a Covariate in Analysis? n (%)** |  |  |  |  |  |  |  |  |  |
| Yes | 25 (4) | 16 (5) | 6 (5) | 1 (1) | 1 (1) | 1 (3) | - | - | - |
| **Reported Disability? n (%)** |  |  |  |  |  |  |  |  |  |
| Yes | 156 (22) | 81 (26) | 19 (16) | 25 (27) | 10 (11) | 6 (15) | 6 (32) | 6 (46) | 3 (30) |
| **Reported Neurodiversity?**  **n (%)** |  |  |  |  |  |  |  |  |  |
| Yes | 36 (5) | 16 (5) | 9 (8) | 5 (5) | 3 (3) | 1 (3) | - | 2 (15) | - |

Notes. White: minimum value (%). Darkest shade of grey: maximum value (%). Values in between are indicated by the gradient. LGBTQ: Lesbian, Queer, Bisexual, Transgender, and Queer. Questioning: Individuals who were questioning their gender identity. TGD: Transgender and Gender-Diverse. ***:** Percentages add up to more than 100% and n adds up to more than total number of studies due to multiple age groups and/or racial/ethnic groups being represented in some studies.

**Table 2. Summary of study characteristics of studies cited in chapter 12, titled “Hormone Therapy”.**

|  | All  n = 115 (100%) | Cross Sectional  n = 39 (34%) | Prospective Cohort  n = 29 (25%) | Retrospective Cohort  n = 19 (17%) | Qualitative  n = 4 (3%) | Mixed-Methods  n = 8 (7%) | Randomized Controlled Trial  n = 9 (8%) | Case Control  n = 5 (4%) | Pre-Post  n = 2 (2%) |
| --- | --- | --- | --- | --- | --- | --- | --- | --- | --- |
| **Median Year**  **(Range)** | 2018  (1981 –2022) | 2017  (2000 – 2021) | 2018  (1981 – 2022) | 2019  (1997 – 2021) | 2018  (2015 – 2020) | 2014  (2008 – 2021) | 2013  (2003 – 2021) | 2014  (2007 – 2019) | 2019.5  (2019 – 2020) |
| **Gender Identities**  **n (%)** |  | | | | | | | | |
| Cisgender | 9 (8) | 1 (3) | 2 (7) | 1 (5) | - | - | 2 (22) | 3 (60) | - |
| Intersex | - | - | - | - | - | - | - | - | - |
| Eunuch | - | - | - | - | - | - | - | - | - |
| LGBTQ | 2 (2) | - | - | - | 1 (25) | 1 (12) | - | - | - |
| Not Reported | 4 (3) | 2 (5) | 2 (7) | - | - | - | - | - | - |
| Questioning | 8 (7) | 1 (3) | 5 (17) | 1 (5) | - | 1 (12) | - | - | - |
| TGD + Cisgender | 9 (8) | 5 (13) | 2 (7) | - | - | 1 (12) | - | 1 (20) | - |
| TGD | 83 (72) | 30 (77) | 18 (62) | 17 (90) | 3 (75) | 5 (63) | 7 (78) | 1 (20) | 2 (100) |
| **Race / Ethnicity**  **n (%) *** |  | | | | | | | | |
| Asian or Pacific Islander | 32 (28) | 14 (36) | 4 (14) | 5 (26) | 2 (50) | 2 (25) | 3 (33) | 1 (20) | 1 (50) |
| Black or African Descent | 35 (30) | 16 (41) | 5 (17) | 4 (21) | 2 (50) | 2 (25) | 4 (44) | 1 (20) | 1 (50) |
| Hispanic or Latinx | 30 (26) | 13 (33) | 4 (14) | 5 (26) | 2 (50) | 2 (25) | 3 (33) | - | 1 (50) |
| Indigenous or Aboriginal | 17 (15) | 9 (23) | 3 (10) | - | 1 (25) | 1 (13) | 2 (22) | - | 1 (50) |
| Multiple Races/Ethnicities | 19 (17) | 11 (28) | 2 (7) | 1 (5) | 3 (75) | 1 (13) | - | - | 1 (50) |
| Not Disclosed or Unknown | 28 (24) | 10 (26) | 5 (17) | 6 (32) | - | 2 (29) | 4 (44) | 1 (20) | - |
| Not reported | 65 (57) | 19 (49) | 20 (69) | 13 (68) | - | 5 (63) | 4 (44) | 3 (60) | 1 (50) |
| Other | 26 (23) | 13 (33) | 5 (17) | 3 (16) | 1 (25) | - | 3 (33) | 1 (20) | - |
| White | 48 (42) | 19 (49) | 8 (28) | 6 (32) | 4 (100) | 3 (38) | 5 (56) | 2 (40) | 1 (50) |
| **Age Groups Represented**  **n (Years; %) *** |  | | | | | | | | |
| 12 ≤ age | 11 (10) | 3 (8) | 5 (13) | 3 (16) | - | - | - | - | - |
| 13 ≤ age ≤ 17 | 30 (26) | 11 (28) | 8 (21) | 6 (32) | 1 (25) | 2 (25) | - | 1 (20) | 1 (50) |
| 18 ≤ age ≤ 29 | 72 (63) | 23 (59) | 19 (49) | 13 (68) | 3 (75) | 5 (63) | 6 (67) | 2 (40) | 1 (50) |
| 30 ≤ age ≤ 50 | 67 (58) | 21 (54) | 15 (38) | 12 (63) | 2 (50) | 3 (38) | 9 (100) | 5 (100) | - |
| 51 ≥ age | 41 (36) | 16 (41) | 6 (15) | 7 (37) | 2 (50) | 1 (13) | 5 (56) | 4 (80) | - |
| **Stratification by Gender? n (%)** |  |  |  |  |  |  |  |  |  |
| Yes | 45 (39) | 12 (31) | 15 (52) | 10 (53) | - | 3 (38) | 2 (22) | 2 (40) | 1 (50) |
| **Gender Included as a Covariate in Analysis? n (%)** |  |  |  |  |  |  |  |  |  |
| Yes | 2 (2) | 1 (3) | - | - | - | - | - | 1 (20) | - |
| **Stratification by Sex? n (%)** |  |  |  |  |  |  |  |  |  |
| Yes | 55 (48) | 17 (44) | 18 (62) | 10 (34) | - | 3 (38) | 3 (33) | 2 (40) | 2 (100) |
| **Sex Included as a Covariate in Analysis? n (%)** |  |  |  |  |  |  |  |  |  |
| Yes | 2 (2) | 2 (5) | - | - | - | - | - | - | - |
| **Reported Disability? n (%)** |  |  |  |  |  |  |  |  |  |
| Yes | 37 (32) | 14 (36) | 6 (21) | 6 (32) | - | 4 (50) | 4 (44) | 3 (67) | - |
| **Reported Neurodiversity? n (%)** |  |  |  |  |  |  |  |  |  |
| Yes | 7 (6) | 4 (10) | 3 (10) | - | - | - | - | - | - |

Notes. White: minimum value (%). Darkest shade of grey: maximum value (%). Values in between are indicated by the gradient. LGBTQ: Lesbian, Queer, Bisexual, Transgender, and Queer. Questioning: Individuals who were questioning their gender identity. TGD: Transgender and Gender-Diverse. ***:** Percentages add up to more than 100% and n adds up to more than total number of studies due to multiple age groups and/or racial/ethnic groups being represented in some studies.

**C6. I applaud the authors for this inclusion.**

R6. We thank the Reviewer for this comment for our statement on lines 213 to 219:

“Underrepresentation of older adults in clinical research is widespread across virtually all fields (26-32) with resulting care guidelines often relying on studies with limited older participants, potentially reducing their applicability in this age group (33-35). A recent review (36) outlined common barriers and potential solutions to increase participation of older adults in clinical research, which coupled with TGD-inclusive practices (37, 38) could lead to a greater evidence base in the health care of older adults.”

**C7. I think this would be stronger if grounded in the results section. Specifically commenting on number of 'longitudinal studies' (I think you would class prospective and RCT) together, and how this changes over time. Were there any long term cohorts cited at all? E.g. ENIGI**

R7. Thank you for this comment. We conducted a similar analysis to the one investigating temporal patterns in the gender composition of study populations, and have included the results as Figure 3. For this analysis we compared temporal patterns in 4 study designs: 1) cross sectional studies, 2) longitudinal studies (includes prospective and retrospective cohort studies, randomized controlled trials, case control, and pre-post studies), 3) qualitative studies, 4) mixed methods studies. Overall, we found that there was a significant decrease in longitudinal and increase in qualitative studies from 1969 to 2023. Cross-sectional and mixed method studies demonstrated no significant change in trends. Please see pages 12 and 13, lines 172-77, for the updated results.

“From 1969 to 2023, we observed a change in the composition of study designs. Longitudinal studies were initially most common, with a temporal increase in cross sectional, qualitative and mixed method studies (Fig 3A). Moreover, the proportion of longitudinal studies decreased (p=0.04) while the proportion of qualitative studies increased (p=0.03; Fig 3B). The proportions of cross sectional (p=0.09) or mixed-methods (p=0.80) studies did not change over the same time period.”

Please see below for Figure 3A and 3B, and the included figure legend on pages 13, lines 178 to 184:

**Fig 3. Temporal patterns in study designs among studies cited in the WPATH SOC 8**. A) Absolute number of studies published and study designs as a function of time. B) Design of studies over time expressed as percentage. P_Trend_ calculated for the proportion of studies involving exclusively TGD individuals, not specifying gender identity, both TGD and cisgender individuals, and exclusively cisgender individuals. P_Trend_: p-value indicating if there is a significant change in trend over the time period. Longitudinal studies consisted of prospective and retrospective cohort studies, randomized controlled trials, case control, and pre-post studies.”

**C8. Also prospectively planning for this. See SAGER guidelines, NIH guidance, MESSAGE policy.**

R8. We thank the Reviewer for this comment.

Please see page 20, lines 273-278:

“At a time when TGD health research is under scrutiny, and in alignment with journal (5), funding agencies (6-8) and policy framework (9) guidance on the inclusion of sex and gender in research, prospectively planning for both inclusion and disaggregation of sex and gender is required to support sex- and gender-based analyses of not only TGD health research, but all health research.”

**C9. More broadly, I think the authors would do well to expand the analysis in order to draw stronger conclusions.**

**WPATH recommendations have a number of restrictions on how they are drawn up, including the need to be globally applicable. Although broader literature can be cited in the text, the restrictions on the recommendations, along with word count, may limit the citing of 'state of the art research'. This is worth acknowledging.**

**For this reason, it may be useful to compare SOC8 studies longitudinally but to the previous SOC7.**

**Further, the authors have a specific endpoint around studies related hormone therapy. It would be helpful to contrast this sub analysis to the remainder of the literature (is it more or less robust)? And also to the Endocrine Society guideline references, as the authors explicitly state these have over-reliance on studies with cisgender participants only.**

**Although these do constitute significant further analysis they would add weight to conclusions on the direction of the field and point to a need for more frequent guideline review.**

R9. We thank the Reviewer for these comments. We have included discussion of how WPATH SOC 8 guidelines were developed in the limitations section of the discussion on page 20, lines 279-286:

“This study has limitations. First, only studies referenced in the WPATH SOC 8 were included, rather than the entire body of literature informing TGD healthcare. However, WPATH is an international and multidisciplinary professional association whose mission is to promote evidence-based care of TGD persons; one of the main functions of WPATH is to promote the highest standards of health care for TGD persons through the Standards of Care (10) “based on the best available science”. While important literature may have been missed, the methods used to develop WPATH SOC 8 are publicly available (11) and align with existing frameworks for the development of clinical practice guidelines (12).”

While we agree that a comparison of literature cited in SOC7 and the Endocrinology Society Guidelines compared to SOC8 would be of interest, the objective of this work was to describe the state of the current literature informing care of TGD persons. As such, we have elected to restrict our analysis to the literature informing SOC 8 and hope the Reviewer appreciates our rationale for doing so.

**C12. At a time when literature on TGD health is under scrutiny, the authors may also wish to acknowledge this and suggest pragmatic ways forward that best serve the needs of the population.**

R12. We thank the Reviewer for this comment. We have endeavoured to address this important point on page 20, lines 272 to 278:

“The low proportions of studies incorporating sex and gender identity in analysis of results are in keeping with previous studies (13-15). At a time when TGD health research is under scrutiny, and in alignment with journal (5), funding agencies (6-8) and policy framework (9) guidance on the inclusion of sex and gender in research, prospectively planning for both inclusion and disaggregation of sex and gender is required to support sex- and gender-based analyses of not only TGD health research, but all health research.”

**Reviewer #2: 

Overall impressions:**

**C1. This is a reasonable paper with appropriate methods to answer the questions posed in the introduction. A bibliometric analysis is well conducted (though I am not an expert in this method). The conclusions are a bit broad for the data set used (Literature supporting TGD care) as it over-reaches in its claim that these results possibly demonstrate an increase in inclusion of TDG people in health research in general, which is implied in the conclusion. This was an analysis of the TGD literature in a guideline that was focused on best practices for TGD care, so it is expected to contain primary studies with this population and is not a very novel finding. It does make good points about the type of literature and demographic gaps in the knowledge base that will inform future studies. The analysis of the hormonal care section is sound, though there is a missed opportunity to include analysis of other sections in a similar manner.**

R1. We appreciate the Reviewer’s comments. We aimed to describe the literature included in all of the WPATH SOC 8 guidelines informing TGD health care, and we repeated our analysis of chapter 12, titled *Hormone Therapy*, given the recent heightened focus on this area of TGD health care. It is worth noting that although we did not present the results of all other chapters in the main manuscript, data on chapters 1 to 18 (excluding chapter 12) can be found in Supplementary File 1.

Page 7, lines 143-144:

“Data for individual chapters, excluding Chapter 12, are listed in S1 File.”

**Introduction:**

**C2. Clear rational for the study, given the underrepresented transgender populations and gender in general in scientific studies.**

R2. We thank the Reviewer for this comment.

**C3. 79 The secondary objective is vague in the introduction, with no indication of what they want to explore about gender-affirming hormone therapy guidelines. It is clear later in the article that they are applying the same bibliometric analysis to hormone therapy guidelines, so this should be explicit in the objectives. Also, they presented data only on hormone therapy guidance, not surgical or other care, which are an important part of the guidelines. If this was the only portion of the guidelines that was important it should be explicitly stated in the objectives and give us a reason why surgical guidelines, fertility preservation, sexual and mental health etc. were not included.**

R3. We appreciate the Reviewer’s comments. As we have outlined in an earlier response (R1.), we aimed to describe the literature included in all of the WPATH SOC 8 guidelines informing TGD health care, and we repeated our analysis of chapter 12, titled *Hormone Therapy*, given the recent heightened focus on this area of TGD health care. We agree that other aspects of care are of utmost importance, and as such, our analyses are included supplemental files which include surgical guidelines, fertility preservation, sexual and mental health. We have revised our manuscript to reflect the rationale around this on page 5, lines 82 to 86:

“Our primary objective was to describe the participant demographics and study design of the literature cited in WPATH SOC 8 informing the care of TGD persons. Our secondary objective was to specifically describe the literature informing gender-affirming hormone therapy guidelines, given recent heightened focus on this area of TGD healthcare.”

It is worth noting that although we did not present the results of all other chapters in the main manuscript, data on chapters 1 to 18 (excluding chapter 12) can be found in Supplementary File 1.

Page 7, lines 143-144:

“Data for individual chapters, excluding Chapter 12, are listed in S1 File.”

**Methods:**

**C4. 74 A bibliometric review appears to be a straightforward investigation method to answer the study question about trends in gender based research.**

R4. We thank the Reviewer for this comment.

**C5. 89 Inclusion criteria is appropriate, and the authors should be commended on including non-English studies by sending them through Google Translate. It would be helpful to see a reference that details the accuracy of Google Translate for scientific studies.**

R5. We thank the Reviewer for this comment. We have updated lines 99 to 101 on page 6 to include a reference that details the accuracy of Google Translate:

“Articles in languages other than English were translated to English using Google Translate (1), which has been previously validated in systematic reviews (2).”

**C6. 96 Extraction characteristics appear appropriate for a bibliometric analysis.**

R6. We thank the Reviewer for this comment.

**C7. 104 With straight forward data extraction, and little subjectivity, I would expect the agreement between reviewers to be higher. There is no mention of inter-rater reliability statistic and how it was generated for the quality assurance portion of the review, though I like the thoroughness of including this QA. Consider including more detail about how this was calculated or if it was ad hoc.**

R7. We thank the Reviewer for this comment. We have included more information on page 6, lines 112 to 116:

“Agreement between the two reviewers (BSBP, NG) was calculated by dividing the number of articles where the abstracted data was identical between reviewers by the total number of articles assessed. Abstracted data was identical in 70% of articles and 30% of articles had at least one discrepancy, which was resolved by a third reviewer (SBA).”

**C8. 110 Linear-by-linear trend test appears to be an appropriate statistic to uncover trends in the temporal data.**

R8. We thank the Reviewer for this comment.

**Results:**

**C9. Well reported, clearly written, tables are well laid out and easy to interpret.**

R9. We thank the Reviewer for this comment.

**C10. Figure 1 has spelling error “sough” appears to be “sought”.**

R10. We thank the Reviewer for this comment. The spelling error has been corrected:

**C11. To make it clear that the exclusion criteria were very specific and broad, adding a note in methods to see figure 1 for complete list of exclusion criteria would be helpful, there were many more categories than expected based on the methods described in the methods section.**

R11. Thank you for this comment. We have included a sentence in line 99 of page 6 for readers to see Figure 1 for the complete list of exclusion criteria:

“Fig 1 includes the complete list of exclusion reasons.”

**C12. Figure 2 is clear, but does not add much beyond the table, just a re-representation of the data in visual format. Authors could consider leaving this out, unless the editors feel it further clarifies the findings in table 1.**

R12. We thank the Reviewer for this comment. Figure 2 has now been removed from the manuscript.

**C13. Figure 3: Temporal patterns graphic is very pixilated, this may be the packaging software that collated the document, if not then a higher res graphic would be needed. Cannot interpret the hashed sections effectively and had to guess.**

R13. Thank you for this comment. This was due to how the software collated the document. All figures have been uploaded as TIFF files as per journal submission guidelines. We have now also uploaded the figures as PDF documents to ensure figures can be interpreted.

**C14. 148-153 It is not unexpected that a data set that included primary literature that was informing the care of TGD populations to have a higher percentage of TGD participants. See note below.**

R14. We appreciate the Reviewer’s comment. We have included this limitation on pages 20 and 21, lines 293 to 297:

“It is also important to note the potential bias toward studies with more TGD populations. It should not be unexpected that a data set including studies informing the care of the TGD population have a high proportion of TGD study participants. However, literature used to inform guidelines has been previously shown to not be inclusive of sex and gender, as well as other intersectional, considerations (15-21).”

**C15. 157 PTrend is not defined in the fig 3 caption.**

R15. Thank you for this comment. We have updated the figure legend of (what is now) Figure 2 to define P_Trend_ (Page 12, lines 161 to 171):

“Fig 2. Temporal patterns in the gender composition of study populations among studies cited in the WPATH SOC 8. A) Absolute number of studies published and gender identity of participants as a function of time. B) Gender identity of participants involved in studies over time expressed as percentage. P_Trend_ calculated for the proportion of studies involving exclusively TGD individuals, not specifying gender identity, both TGD and cisgender individuals, and exclusively cisgender individuals. P_Trend_: p-value indicating if there is a significant change in trend over the time period. TGD: Transgender and Gender-diverse. Given terms including eunuch, intersex, LGBTQ, do not provide sufficient information to categorize an individual’s gender identity, they were included in the “Not Specified” group. Participants who were questioning their gender identity were included in the “TGD” group.”

**Discussion:**

**C16. 88 The conclusion that these results “suggest a change towards improved inclusion of methods allowing for greater representation of a diversity of participants within health research” is not supported by this study. This study looked at TGD associated research only, I suspect that TGD populations are still very unrepresented in health research in general, and this study did not look beyond the literature informing TDG care. Suggest a revision to narrow the scope of this conclusion.**

R16. We have endeavoured to address this important comment in our revised manuscript.

Please see page 17, lines 213-217:

“These results suggest improved methods with greater and more diverse participant representation within TGD-associated health research leading to an enriched evidence base informing the care of TGD persons. However, much work still remains in both TGD-associated health research, and health research at large (1).”

Pages 20, lines 272 to 278:

“The low proportions of studies incorporating sex and gender identity in analysis of results are in keeping with previous studies (13-15). At a time when TGD health research is under scrutiny and in alignment with journal (5), funding agencies (6-8) and policy framework (9) guidance on the inclusion of sex and gender in research, prospectively planning for both inclusion and disaggregation of sex and gender is required to support sex- and gender-based analyses of not only TGD health research, but all health research.”

**C17. 198 The secondary outcome focusing on hormone therapy seems to be a more important finding, in that the guidance is somewhat based on hypogonadal (cisgender?) individuals.**

R17. We appreciate the Reviewer’s comment, which we have aimed to address on page 17, lines 212 to 217:

“5) hormone therapy guidelines are largely informed by studies including TGD participants."

**C18. 207-219 Excellent observation re: age representation and need for research that captures longitudinal data, this is one of the major findings and informs future research design.**

R18. We thank the Reviewer for this comment.

**C19. 248 Limitations are well laid out. The above limitation and possibility of bias toward studies with more TGD populations may be included here.**

R19. We appreciate the Reviewer’s comment. We have included this limitation on pages 20 and 21, lines 293 to 297:

“It is also important to note the potential bias toward studies with more TGD populations. It should not be unexpected that a data set including studies informing the care of the TGD population have a high proportion of TGD study participants. However, literature used to inform guidelines has been previously shown to not be inclusive of sex and gender, as well as other intersectional, considerations (15-21).”

**References**

1. Google. Google Translate 2006 [Available from: https://translate.google.ca].

2. Jackson JL, Kuriyama A, Anton A, Choi A, Fournier J-P, Geier A-K, et al. The Accuracy of Google Translate for Abstracting Data From Non–English-Language Trials for Systematic Reviews. Annals of Internal Medicine. 2019;171(9):677-9.

3. World Health Organization. International classification of functioning, disability and health : ICF. Geneva: World Health Organization; 2001.

4. Chapman R. Defining neurodiversity for research and practice. 2020. p. 218-20.

5. Heidari S, Babor TF, De Castro P, Tort S, Curno M. Sex and Gender Equity in Research: rationale for the SAGER guidelines and recommended use. Research Integrity and Peer Review. 2016;1(1):2.

6. Canadian Institutes of Health Research. How to integrate sex and gender into research 2019 [Available from: https://cihr-irsc.gc.ca/e/50836.html].

7.National Institutes of Health: Center for Scientific Review. Premise, Rigor, Sex as a Biological Variable 2020 [Available from: https://public.csr.nih.gov/FAQs/ReviewersFAQs/PremiseRigorSexBiologicalVariable#7].

8. European Commission. Gender in EU research and innovation 2023 [Available from: https://rea.ec.europa.eu/gender-eu-research-and-innovation_en].

9. Medical Science Sex and Gender Equity. About MESSAGE 2023 [Available from: https://www.messageproject.co.uk/about/].

10. Coleman E, Radix AE, Bouman WP, Brown GR, de Vries ALC, Deutsch MB, et al. Standards of Care for the Health of Transgender and Gender Diverse People, Version 8. Int J Transgend Health. 2022;23(Suppl 1):S1-s259.

11. World Professional Association of Transgender Health. Methodology for the Development of SOC8 2022 [Available from: https://www.wpath.org/soc8/Methodology].

12. Murad MH. Clinical Practice Guidelines: A Primer on Development and Dissemination. Mayo Clin Proc. 2017;92(3):423-33.

13. Scheim A, Kacholia V, Logie C, Chakrapani V, Ranade K, Gupta S. Health of transgender men in low-income and middle-income countries: a scoping review. BMJ Glob Health. 2020;5(11).

14. Marshall Z, Welch V, Minichiello A, Swab M, Brunger F, Kaposy C. Documenting Research with Transgender, Nonbinary, and Other Gender Diverse (Trans) Individuals and Communities: Introducing the Global Trans Research Evidence Map. Transgend Health. 2019;4(1):68-80.

15. Gulamhusein N, Turino Miranda K, Dumanski SM, González Bedat MC, Ulasi I, Conjeevaram A, et al. Sex‐ and Gender‐Based Reporting in Antihypertensive Medication Literature Informing Hypertension Guidelines. Journal of the American Heart Association. 2024;0(0):e030613.

16. Hassan R, Riehl-Tonn VJ, Dumanski SM, Lyons KJ, Ahmed SB. Female sex-specific and -predominant cardiovascular risk factors and heart failure practice guidelines. Am Heart J. 2022;247:63-7.

17. Fahmawi S, Schinke C, Thanendrarajan S, Zangari M, Shaughnessy JD, Jr., Zhan F, et al. Under-representation of black patients with multiple myeloma in studies supporting International Myeloma Working Group guidelines. J Cancer Policy. 2023;37:100433.

18. Nazer L, Abusara A, Aloran B, Szakmany T, Nabulsi H, Petushkov A, et al. Patient diversity and author representation in clinical studies supporting the Surviving Sepsis Campaign guidelines for management of sepsis and septic shock 2021: a systematic review of citations. BMC Infectious Diseases. 2023;23(1):751.

19. Lubarsky R, Ambinder D, Barnett J, Choudhury M, Saji A, Fishman AI, et al. Prostate Cancer: Under-representation of African American Men in Research Studies Used in the Latest NCCN Guidelines. Urology. 2023;180:28-34.

20. Zuberi SA, Burdine L, Dong J, Feuerstein JD. Representation of Racial Minorities in the United States Colonoscopy Surveillance Interval Guidelines. J Clin Gastroenterol. 2023.

21. Williamson TJ, Battaglia PJ, Gliedt JA, Spector AL, Williams JS. Trials Informing Back Pain Guidelines Underreport Key Sociodemographic Data. J Health Care Poor Underserved. 2023;34(1):357-76.
