# Supplementary material for: Characterization of the literature informing health care of transgender and gender-diverse persons: A bibliometric analysis
Source: PLoS One. 2024 Oct 4;19(10):e0309169. doi: 10.1371/journal.pone.0309169 (PMC11452042; doi:10.1371/journal.pone.0309169)
Supplement: Attachment — Submitted filename: Response to Reviewers.docx [file pone.0309169.s005.docx]

August 3, 2024

Emily Chenette, PhD 
Editor-in-Chief 
PLOS ONE

**Manuscript ID#: *PONE-D-24-13832.R2***

**Title:** Characterization of the literature informing health care of transgender and gender-diverse persons: A bibliometric analysis

Thank you for allowing us the opportunity to resubmit our manuscript, based on the thorough review and thoughtful comments provided by the Reviewers. We feel that we have been able to satisfactorily address all the comments raised, which has further enhanced the quality of the manuscript. We have provided an itemized summary of the changes made to the paper below with the Reviewers’ comments provided in bold, followed by our responses. We have provided a tracked changed version of the manuscript, as well as a version without track changes.

**Reviewer #1:**

**C1. The authors should acknowledge in limitations the use of Google Translate for studies not in English and provide the N number here.**

R1. We thank the Reviewer for this comment. We have included the total number of non-English studies that were translated on page 6, lines 99 – 101:

“Articles in languages other than English (n=5) were translated to English using Google Translate (1), which has been previously validated in systematic reviews (2).”

We have also acknowledged this as a limitation of our study on page 21, lines 308 – 313:

“Moreover, articles in languages other than English were translated using Google Translate (1) into English, rather than having a native language speaker review the studies, potentially impacting the accuracy of our review. Nevertheless, a previous study has validated the use of this translation tool in systematic reviews, demonstrating an 85-97% accuracy when compared to native language speakers (2).”

**C2. The authors have helpfully commented on inclusion of neurodiverse participants in the Results, this deserves a few lines in the discussion, in the same way as for older adults, as it is a significant proportion of the gender diverse population.**

R2. We appreciate the Reviewer’s comment. We have updated lines 243 – 251 on pages 18 and 19 to include a discussion of neurodivergent study participants:

“Furthermore, very few studies reported participant disability or neurodiversity status. Previous studies have suggested increased rates of autism, as well as other neurodevelopmental and psychiatric diagnoses in TGD individuals relative to cisgender individuals (3, 4), and thus appropriate representation in study participants is crucial to tailor care meeting the needs of TGD populations. A recent review (5) outlined common barriers and potential solutions to increase participation of older adults in clinical research, which coupled with TGD-inclusive practices (6, 7), could lead to a greater evidence base in the health care of TGD persons.”

**C3. The authors have given the proportion of longitudinal studies in the results and commented on this, which is helpful. I note from the results that the number of qualitative studies has also increased which is important as it suggests as it suggests we MAY be better evaluating patient experience in studies - this deserves a comment in the discussion.**

R3. We thank the Reviewer for this comment. We have included a discussion of the increase in the number of qualitative studies on page 19, lines 258 – 260:

“However, the significant increase in the proportion of qualitative studies is encouraging as it may reflect a shift towards a greater number of studies examining TGD individuals’ experiences.”

**R4. In line 255 the authors discuss RCT as high-quality evidence. It is worth a comment that this may be ethically challenging in this space, given the public discourse around the Cass review in the UK.**

C4. We appreciate the Reviewer’s comment. We have updated lines 265 – 267 on page 19 with the following:

“Of note, RCTs are not always appropriate or ethical, particularly in the absence of clinical equipoise, and observational studies may be more reflective of “real world” data (8).

**Reviewer #2:**

**C1. Thank you for your revision of this article, it will add significantly to the literature on care of TGD populations and keep up the great work!**

R1. We thank the Reviewer and greatly appreciate their comment.

**References**

1. Google. Google Translate 2006 [Available from: https://translate.google.ca].

2. Jackson JL, Kuriyama A, Anton A, Choi A, Fournier J-P, Geier A-K, et al. The Accuracy of Google Translate for Abstracting Data From Non–English-Language Trials for Systematic Reviews. Annals of Internal Medicine. 2019;171(9):677-9.

3.Warrier V, Greenberg DM, Weir E, Buckingham C, Smith P, Lai MC, et al. Elevated rates of autism, other neurodevelopmental and psychiatric diagnoses, and autistic traits in transgender and gender-diverse individuals. Nat Commun. 2020;11(1):3959.

4. Kallitsounaki A, Williams DM. Autism Spectrum Disorder and Gender Dysphoria/Incongruence. A systematic Literature Review and Meta-Analysis. J Autism Dev Disord. 2023;53(8):3103-17.

5. Forsat ND, Palmowski A, Palmowski Y, Boers M, Buttgereit F. Recruitment and Retention of Older People in Clinical Research: A Systematic Literature Review. J Am Geriatr Soc. 2020;68(12):2955-63.

6. Rytz CL, Beach LB, Saad N, Dumanski SM, Collister D, Newbert AM, et al. Improving the inclusion of transgender and nonbinary individuals in the planning, completion, and mobilization of cardiovascular research. American Journal of Physiology-Heart and Circulatory Physiology. 2023;324(3):H366-H72.

7. Owen-Smith AA, Woodyatt C, Sineath RC, Hunkeler EM, Barnwell T, Graham A, et al. Perceptions of Barriers to and Facilitators of Participation in Health Research Among Transgender People. Transgend Health. 2016;1(1):187-96.

8. Toews I, Anglemyer A, Nyirenda JL, Alsaid D, Balduzzi S, Grummich K, et al. Healthcare outcomes assessed with observational study designs compared with those assessed in randomized trials: a meta-epidemiological study. Cochrane Database Syst Rev. 2024;1(1):Mr000034.
